# Evaluation of a Multilevel Program to Improve Clinician Adherence to Management Guidelines for Acute Ischemic Stroke

**DOI:** 10.1001/jamanetworkopen.2022.10596

**Published:** 2022-05-06

**Authors:** Yi Chen, Xiaoxian Gong, Wansi Zhong, Jianbing Wang, Zongming Yang, Shenqiang Yan, Fangli Geng, Ying Zhou, Xuting Zhang, Zhicai Chen, Haitao Hu, Lusha Tong, Hongfang Chen, Shaofa Ke, Yuping He, Yaxian Wang, Xiaoling Zhang, Zhimin Wang, Zhihui Chen, Heng Zhao, Changzheng Yuan, Min Lou

**Affiliations:** 1 Department of Neurology, the Second Affiliated Hospital of Zhejiang University, School of Medicine, Hangzhou, China; 2 Department of Epidemiology and Biostatistics at School of Public Health and National Clinical Research Center for Child Health of the Children's Hospital, Zhejiang University School of Medicine, Hangzhou, China; 3 Health Policy program, Harvard University Graduate School of Arts and Sciences, Cambridge, Massachusetts; 4 Department of Neurology, Jinhua Hospital of Zhejiang University, Jinhua Municipal Central Hospital, Jinhua, China; 5 Department of Neurology, Taizhou Hospital, Wenzhou Medical University, Taizhou, China; 6 Department of Neurology, Zhuji People's Hospital of Zhejiang Province, Shaoxing, China; 7 Department of Neurology, Huzhou Central Hospital, Huzhou, China; 8 Department of Neurology, Jiaxing Second Hospital, Jiaxing, China; 9 Department of Neurology, Taizhou First People's Hospital, Huangyan Hospital of Wenzhou Medical University, Taizhou, China; 10 Department of Neurology, Lanxi People's Hospital, Jinhua, China; 11 Department of Neurosurgery, Stanford University, Stanford, California; 12 School of Public Health, Zhejiang University School of Medicine, Hangzhou, China; 13 Department of Nutrition, Harvard T.H. Chan School of Public Health, Boston, Massachusetts

## Abstract

**Importance:**

Promotion of clinician adherence to stroke guidelines can improve stroke outcomes.

**Objective:**

To investigate the outcomes of a multilevel system program on clinician adherence to guidelines for treatment of patients with acute ischemic stroke (AIS).

**Design, Setting, and Participants:**

This quality improvement study used a prospective interrupted time series (ITS) and difference-in-difference (DID) design, from August 1, 2018, to January 31, 2020, divided into preprogram term and short and long postprogram terms; each term had 6 months. Data were collected during hospitalization and at discharge with an automated medical record data capture system in 58 public hospitals in Zhejiang province, China. Data were analyzed from August 2018 to January 2020.

**Exposures:**

The multilevel system program included a modularized standard template for medical records, centrally supported continuing education, continuous monitoring and feedback, and collaborative workshops.

**Main Outcomes and Measures:**

The primary outcome was adherence to 12 key performance indicators (KPIs), expressed as (1) percentage of patient-applicable KPIs achieved in each participant and (2) percentage of participants among whom all applicable KPIs were achieved (dichotomous all-or-none measure). The secondary outcome was severe disability or death (modified Rankin Scale 5-6) at discharge.

**Results:**

Among 45 091 patients (mean [SD] age, 69 [12] years; 18 347 female [40.7%]), 28 721 from 30 hospitals received the program and 16 370 from 28 hospitals continued routine care. In adjusted DID analysis, the program was associated with an increase in the absolute percentage of KPIs achieved per patient (6.46%; 95% CI, 5.49% to 7.43%), absolute rate of all-or-none success (8.29%; 95% CI, 6.99% to 9.60%), and decreased rate of severe disability or death at discharge (−1.68%; 95% CI, −2.99% to −0.38%). The ITS result showed the program was associated with an increase in KPIs achieved per patient per week (slope change in short-term period, 0.36%; 95% CI, 0.20% to 0.52%; level change in long-term period, (9.64%; 95% CI, 4.58% to 14.69%) and in all-or-none success (slope change in short-term period 0.34%; 95% CI, 0.23% to 0.46%; level change in long-term period 5.89%; 95% CI, 0.19% to 11.59%).

**Conclusions and Relevance:**

The centrally supported program was associated with increases in clinician adherence to guidelines and reduced the proportion of severely disabled or deceased patients with AIS at discharge, providing support for its wider implementation.

## Key Points

**Question:**

Can a multilevel, centrally supported quality program promote clinician adherence to stroke guidelines?

**Findings:**

This quality improvement study included 45 091 patients from 58 hospitals. The multilevel system program was associated with a significant increase in the absolute percentage of key performance indicators achieved per patient per week (adjusted change, 6.46%), the absolute rate of all-or-none success (adjusted change, 8.29%), and a decrease in the rate of severe disability or death at discharge (adjusted change, −1.68%).

**Meaning:**

These findings suggest that this multicenter program may serve as a model for ischemic stroke–related quality improvement activities.

## Teaser

This quality improvement study evaluates the outcomes associated with a program to improve clinician adherence to management guidelines for acute ischemic stroke in hospitals in China.

## Introduction

Stroke has imposed an enormous disease burden on the health care system, especially in developing countries, such as China.^1^ Although evidence-based clinical stroke guidelines provide recommendations for stroke treatments, gaps exist between the guideline recommendations and clinical practice. To enhance clinician adherence to stroke guidelines, the American Heart Association has set a model by initiating the Get With The Guidelines (GWTG)–Stroke program in 2003, which has reshaped stroke care delivery and remarkably improved national stroke care quality in the US^2^ with the monitoring of performance measures, alternatively called key performance indicators (KPIs). Later in 2018, a small improvement in performance measures for acute ischemic stroke (AIS) was also confirmed in China by a cluster randomized clinical trial with a population of 4800 patients.^3^ However, a similar quality improvement program did not result in a significant increase in composite adherence score in another Brazilian clinical trial.^4^ It remains unknown whether quality improvement can be generalized to general population settings in developing countries with large populations, but less well-financed national health systems.

An interrupted time series (ITS) design was conducted through the CASE-Stroke program (Computer-based Online Database of Acute Stroke Patients for Stroke Management Quality Evaluation), which was characterized by a central quality care initiative team^5^ aiming to evaluate whether the implementation of a multilevel, centrally supported quality of care program for hospitalized patients with stroke promotes stroke care quality in China. To clear the major barrier of collecting clinical data, an automated medical record data capture system was developed to routinely obtain the KPIs from the original medical documents of patients with AIS without manual data entry that may lead to the loss of original authenticity. This quality improvement study aimed to set a practical model for developing countries with less well-financed national health systems to improve stroke care quality.

## Methods

### Study Design

The detailed information for the methods is available in the eAppendix in Supplement 1. In brief, the CASE-Stroke study was a general population study (ClinicalTrials.gov identifier, NCT03684629). The current quality improvement study was approved by the local ethics committee and followed the Standards for Quality Improvement Reporting Excellence (SQUIRE) reporting guideline for quality improvement studies. Written informed consent was obtained at the hospital levels. Because patient information was deidentified and anonymized before being released to the researchers, the informed consent requirement was waived by the institutional review board. The study period included 2 stages: the preprogram term, from August 1, 2018, to January 31, 2019, and the postprogram term, from February 1, 2019, to January 31, 2020. Considering that the outcomes of the program may take time to manifest,^6^ we divided the postprogram term into a short-term period from February 1, 2019, to July 31, 2019, and a long-term period from August 1, 2019, to January 31, 2020.

### Hospitals and Patients

All hospitals in the CASE-II registry volunteered to participate in this study, then we conducted a prerandomization survey and randomly selected 30 hospitals to join the program group. Among the remaining hospitals, 28 were selected for the nonequivalent control (nonprogram) group after matching hospital characteristics by propensity score matching. Two hospitals refused to participate in this study because they were unable to participate in monthly video conferencing. Finally, we enrolled 30 hospitals in the program group and 28 hospitals in the nonprogram group. Patients were eligible if they were aged 18 years or older with AIS confirmed by brain computed tomography or magnetic resonance imaging within 7 days after symptom onset and admitted to wards directly or through the emergency department. Patients with other cerebrovascular diseases, such as cerebral hemorrhage and cerebral venous sinus thrombosis, were excluded.

### Data Collection

An innovative automated medical record data capture system was used to collect the data of stroke care quality in each hospital. The original medical documents were saved as portable document formats or images, then recognized, preprocessed, and sent to multiple optical character recognition engines to build documents with recognized text (eFigure 1 in Supplement 1). Range checks were used to check for out-of-range data and prompted the trained study investigators to correct or review the data collection outside of the predefined range.

### Multilevel System Program 

The program was designed as an online, interactive, sustainable, and modularized training program by a panel of stroke experts according to evidence-based guidelines (eAppendix and eFigure 2 in Supplement 1), and a professional medical central quality care initiative (QCI) team implemented the program with neurologists, including a modularized standard template for medical records and centrally supported continuing education, continuous monitoring and feedback, and collaborative workshops via monthly video conferencing.

Clinicians integrated the evaluation plan and prescription plan involving the predefined performance measures according to the consensus statements and guidelines into admission records and discharge records through the embedded modularized standard template. The corresponding care plan for each eligible patient with stroke was then followed. Centrally supported continuing education required all neurologists from the program hospitals to attend the monthly video conference, aiming to educate them on the guideline recommendations and quality improvement–related studies, and then increase their belief in the benefit of engaging them on the quality improvement process. In addition, the QCI team used a real-time KPI presentation system and feedback reports on performance to achieve continuous monitoring and feedback and encourage the hospital personnel to seek continuous improvement. The QCI team required the clinicians to display the performance feedback reports as a slide for discussion on the monthly video conference. After the feedback report, the QCI team organized a collaborative workshop to seek potential solutions. The clinicians shared their problems and the possible causes, and the experts shared tools and ideas that had been developed, sought potential solutions, and helped to tailor the implementations to improve the needs of each hospital. Then each hospital developed a simple improvement plan for the following month to ensure the implementation of all KPIs.

### Measures

KPIs are the objective quantitative management indicators that facilitate adherence by the hospital. The primary outcomes were clinician adherence to the 12 predefined evidence-based KPIs, as detailed in eTable 1 in Supplement 1, expressed as a composite measure and an all-or-none measure as coprimary outcomes. The composite measure is defined as the total number of eligible KPIs divided by total number of KPIs implemented for each eligible patient. The all-or-none score is defined as the proportion of eligible patients for whom all of the KPIs were performed.^7^ In addition, the percentage of each individual KPI was also calculated as the total number of patients divided by the total number of KPIs performed for eligible patients. The KPI of intravenous recombinant tissue-type plasminogen activator (rt-PA) was calculated as the total number of patients receiving rt-PA treatment divided by the total number of patients within 7 days of symptom onset, as the number of patients eligible for rt-PA treatment within the 4.5-hour therapeutic time window was not available from participants. The secondary outcomes were the distribution of modified Rankin Scale (mRS) score at discharge and severe disability or death, which was defined as mRS score 5 to 6.

### Statistical Analysis

Baseline characteristics of hospitals and patients were compared between the program and nonprogram groups. An interrupted time series (ITS) model was used to evaluate the outcome of the program on the KPIs. A control (nonprogram) group was included to further control time-varying confounders.^8,9^ Data from the preprogram period were also included to control for biases at the baseline level and linear trend (linear slope). The rapid change of rates in KPIs was observed in the short-term period of the program and the gradual change in KPIs was observed over the long-term period, which were defined as slope change and level change, respectively. Segmented linear regression models were used to estimate the changes in levels and linear trends after the implementation of the program.

Multiple imputation (5 times) was performed for missing values. Only National Institute of Health Stroke scale (NIHSS) and low-density lipoprotein cholesterol had missing data at 3.8% (1734 participants) and 10.2% (4621 participants), respectively. A difference-in-differences (DID) analysis was further used to compare changes in outcomes that occurred over time in the program group to the changes in outcomes from the nonprogram group. The model includes the outcomes in 2 periods, preprogram and postprogram. To account for selection bias, the DID model allowed the use of propensity score weighting to match characteristics of both hospitals and patients between the program and nonprogram groups. Characteristics (hospital grade, stroke unit, neurologist available at emergency department, number of stroke physicians, intravenous thrombolysis per year, annual stroke admission, and hypertension) that were not well matched for propensity score weighting were further adjusted in the DID regression analysis.

Sensitivity analyses were performed by repeating the segmented regression models only in the tertiary hospitals and secondary hospitals, respectively. The significance level was set at 5% as 2-sided and *P* < .05. Analyses were conducted using SPSS statistical software version 24.0 (IBM) and R statistical software version 4.0.1 (R Project for Statistical Computing). Data were analyzed from August 2018 to January 2020.

## Results

### Hospital and Patient Characteristics

This study included 30 hospitals in the program group and 28 hospitals in the nonprogram group. Baseline hospital characteristics between the program and nonprogram group were comparable. The analytical population comprised 45 091 patients, with 28 721 (41.5% women) in the program group and 16 370 (41.5% women) in the nonprogram group. Of the included patients, mean (SD) age was 69 (12) years, 18 347 (40.7%) were female, and median baseline NIHSS was 3 (on a scale of 1-5). Baseline patient characteristics are generally similar between the 2 groups, except that the variables of hypertension, low-density lipoprotein cholesterol value, and baseline NIHSS score were slightly higher in the program group (Table 1).

**Table 1.** Baseline Characteristics of Hospitals and Patients in the Program and Nonprogram Group

Characteristic	No. (%)		*P* value
	Program group	Nonprogram group	
Hospital characteristics			
Hospital, No.	30	28	NA
Hospital grade			.39
Tertiary	23 (76.7)	18 (64.3)	
Secondary	7 (23.3)	10 (35.7)	
Teaching hospital	26 (86.7)	21 (75.0)	.33
Stroke unit	26 (86.7)	20 (71.4)	.20
Capacity of hospital, median (IQR), beds			
Hospital department	1000 (800-1550)	800 (600-1295)	.15
Neurology department	54 (47-90)	50 (48-68)	.30
Team of stroke care			
Stroke team on call around the clock	30 (100)	28 (100)	>.99
Brain CT scan available around the clock	30 (100)	28 (100)	>.99
Available with EVT capabilities	24 (80.0)	21 (75.0)	.76
Neurologist available at ED	10 (33.3)	4 (14.3)	.13
Stroke physicians, median (IQR), No.	15 (9-23)	12 (10-15)	.23
Rate of stroke admission in neurology department, median (IQR), %	44 (30-69)	48 (32-58)	.89
Rate of intravenous thrombolysis, median (IQR), %	15 (11-18)	12 (10-15)	.13
Rate of EVT, median (IQR), %	3 (2-7)	1 (0-3)	.06
Professional rehabilitation team	30 (100)	26 (92.9)	.23
Annual stroke admissions, median (IQR), No.	715 (523-978)	362 (206-650)	<.001
Patient characteristics in the preprogram period^a^			
Patients, No.	8116	4952	
Age, mean (SD), y	69.0 (12.4)	69.0 (12.5)	.99
Sex			.96
Female	3327 (41.0)	2033 (41.1)	
Male	4789 (59.0)	2919 (58.9)	
Hypertension	5394 (66.5)	3145 (63.5)	.001
Diabetes	1643 (20.2)	1003 (20.3)	>.99
History of stroke/TIA	1877 (23.1)	1106 (22.3)	.30
Coronary heart disease	392 (4.8)	261 (5.3)	.26
Atrial fibrillation	605 (7.5)	414 (8.4)	.06
Smoking	2715 (33.5)	1588 (32.1)	.10
LDL-C, mean (SD), mg/dL^b^	97.83 (32.87)	95.12 (33.64)	<.001
Baseline NIHSS, median (IQR)	3 (1-6)	3 (1-6)	.02

Abbreviations: CT, computed tomography; ED, emergency department; EVT, endovascular therapy; LDL-C, low-density lipoprotein cholesterol; NA, not applicable; NIHSS, National Institute of Health Stroke Scale; TIA, transient ischemic attack.

SI conversion factor: To convert LDL-C to millimoles per liter, multiply by 0.02586.

^a^ The preprogram period was August 1, 2018, to January 31, 2019.

^b^ To convert to millimoles per liter, multiply by 0.0259.

### Association of Program With the Primary and Secondary Outcomes

The composite measures were increased from 73.2% before program to 80.3% and 85.4% in short-term and long-term periods after program, respectively (eTable 2 and eFigure 3 in Supplement 1). All-or-none scores were increased from 4.6% in the preprogram period to 10.5% and 16.6% in the short-term and long-term periods, respectively. In the nonprogram group, the composite measures were increased from 69.4% before program to 72.6% and 75.8% in the short-term and long-term periods, respectively (eTable 3 and eFigure 3 in Supplement 1). All-or-none scores were increased from 5.4% in the preprogram period to 5.8% and 7.8% in the short-term and long-term periods, respectively. In addition, the proportion of severe disability or death at discharge was reduced after program implementation from 8.7% at baseline to 6.3% and 6.2% in the short-term and long-term periods, respectively. The proportion of severe disability or death at discharge was changed from 4.1% at baseline to 3.6% and 4.0% in the short-term and long-term periods in the nonprogram group, respectively (eTable 2 and eTable 3 in Supplement 1). The changes in individual KPIs are also presented in eTable 2 and eTable 3 in Supplement 1.

### Adherence to Evidence-Based KPIs

The DID analyzed the differential outcomes postprogram between the 2 groups. Characteristics of hospitals and patients in the program and nonprogram group before and after propensity score weighting are shown in eTable 4 in Supplement 1. After weighting and adjusting the confounders of hospital and patient covariates, the program was associated with an increase in the absolute percentage of KPIs achieved per patient (6.46%; 95% CI, 5.49% to 7.43%; *P* < .001), an increase in the absolute rate of all-or-none success (8.29%; 95% CI, 6.99% to 9.60%; *P* < .001), and a decrease in the rate of severe disability or death at discharge (−1.68%; 95% C,I −2.99% to −0.38%; *P* = .01) (Table 2).

**Table 2.** Comparison of Adherence to Evidence-Based KPIs After Program in Patients with Acute Ischemic Stroke Between the Program vs Nonprogram Group

Characteristic	Mean (SD), %				Difference-in-difference	
	Program group		Nonprogram group			
	Preprogram^a^	Postprogram^a^	Preprogram^a^	Postprogram^a^	Estimate (95% CI)^b^	*P* value
Composite measure	71.91 (13.6)	82.54 (9.8)	67.58 (16.7)	73.60 (14.7)	6.46 (5.49 to 7.43)	<.001
All-or-none score	3.91 (14.5)	13.34 (26.1)	4.24 (16.7)	6.55 (20.6)	8.29 (6.99 to 9.60)	<.001
KPIs at the beginning of hospitalization						
NIHSS assessment	84.40 (31.5)	92.54 (21.4)	67.81 (42.5)	71.52 (39.9)	3.03 (0.46 to 5.61)	.02
Intravenous rt-PA	9.77 (24.8)	11.06 (24.9)	9.60 (25.9)	10.27 (26.2)	1.60 (−0.25 to 3.45)	.09
Early antithrombotics	94.88 (18.7)	97.15 (13.1)	96.40 (16.4)	95.86 (17.4)	4.75 (3.45 to 6.06)	<.001
DVT prophylaxis	45.61 (48.0)	57.48 (47.2)	50.21 (48.3)	51.59 (48.0)	12.85 (6.46 to 19.23)	<.001
Dysphagia screening	80.60 (35.5)	95.06 (18.4)	70.65 (41.5)	87.84 (29.1)	1.71 (−0.83 to 4.25)	.19
Rehabilitation evaluation	43.09 (43.6)	79.62 (34.3)	39.12 (44.3)	62.34 (43.1)	19.89 (17.06 to 22.73)	<.001
KPIs at discharge						
Antithrombotics	89.47 (25.5)	95.61 (16.1)	86.70 (29.7)	90.08 (25.5)	4.01 (2.18 to 5.84)	<.001
Antihypertensive medication	64.17 (40.6)	75.02 (35.8)	63.80 (42.9)	63.16 (42.2)	13.0 (9.84 to 16.16)	<.001
Antidiabetic medication	74.61 (40.9)	84.76 (33.0)	72.16 (42.7)	77.02 (39.6)	7.46 (3.14 to 11.78)	.001
Lipid-lowering for LDL-C >100 mg/dL^c^	91.15 (24.4)	95.62 (16.6)	88.07 (28.9)	90.58 (25.5)	3.66 (1.83 to 5.50)	<.001
Anticoagulation for atrial fibrillation	40.47 (47.7)	67.06 (45.9)	43.24 (48.7)	54.31 (48.9)	17.07 (9.58 to 24.56)	<.001
Smoking cessation	97.44 (12.4)	98.41 (9.9)	95.82 (16.8)	95.74 (17.2)	1.13 (0.11 to 2.15)	.03
Clinical outcome at discharge						
Severe disability or death	8.63 (22.5)	6.12 (18.3)	4.54 (18.3)	3.89 (16.5)	−1.68 (−2.99 to −0.38)	.01
Modified Rankin Scale score, mean (SD)	1.85 (1.3)	1.79 (1.1)	1.56 (1.2)	1.58 (1.1)	0.02 (−0.10 to 0.13)	.74

Abbreviations: DVT, deep venous thrombosis; KPI, key performance indicator; LDL-C, low-density lipoprotein cholesterol; NIHSS, National Institutes of Health Stroke Scale; rt-PA, recombinant tissue-type plasminogen activator.

^a^ The preprogram period was August 1, 2018, to January 31, 2019; the postprogram period was February 1, 2019, to January 31, 2020.

^b^ Difference-in-differences for categorical outcomes are shown as percentage points. Data were adjusted for all hospital and patient characteristics in the propensity score weighting analysis. Data were adjusted for unmatched characteristics (hospital grade, stroke unit, neurologist available at emergency department, number of stroke physicians, intravenous thrombolysis per year, annual stroke admission, and hypertension) in the difference-in-differences regression model.

^c^ To convert to millimoles per liter, multiply by 0.0259.

**Figure 1.**
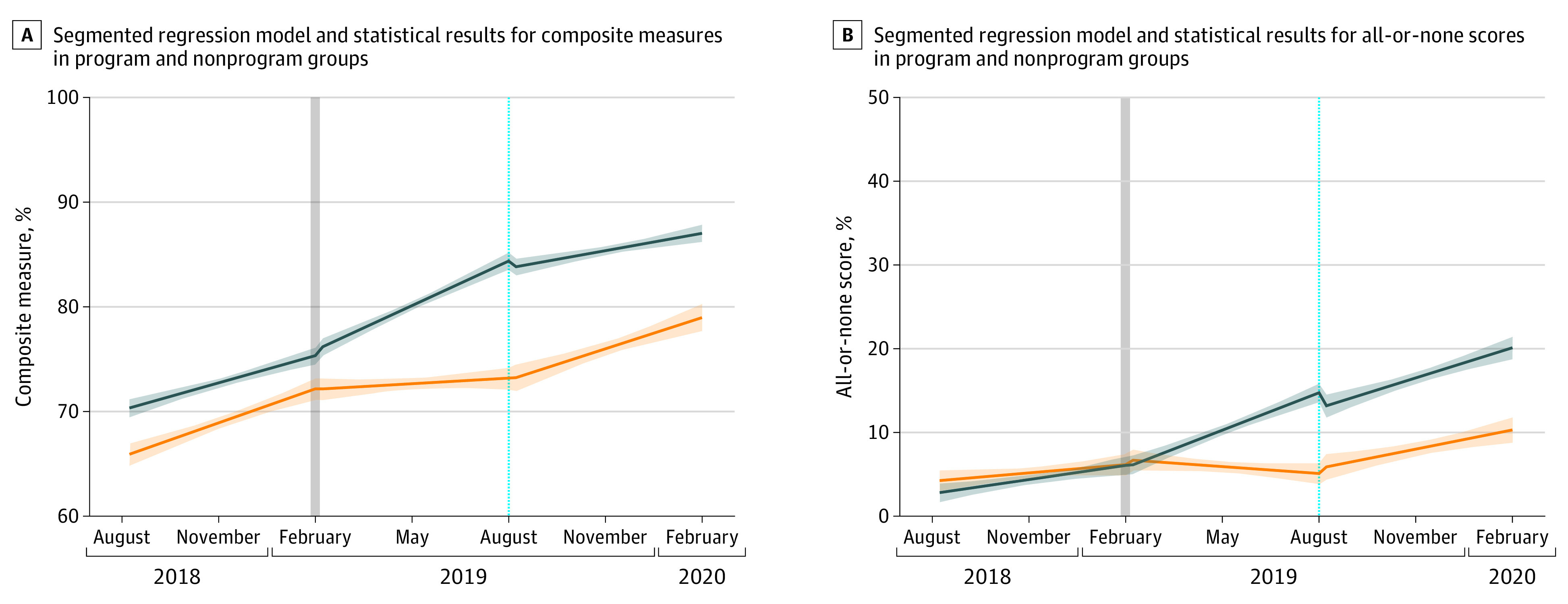
Segmented Regression Model and Statistical Results for the Composite Measures and All-or-None Scores in the Program and Nonprogram Groups The model divided the time course into 3 periods: preprogram, short-term, and long-term postprogram. Shaded regions indicate 95% CIs; vertical lines represent divisions between short-term period and long-term period. Panel A shows the difference between the change in regression line slopes and levels between the program and nonprogram groups for the composite measure. The blue and orange lines indicate the composite measure of the program group and nonprogram group, respectively. Panel B indicates the difference between the changes in slopes and levels in the 2 groups for the all-or-none scores. The blue and orange lines indicate the all-or-none score of the program group and nonprogram group, respectively. Detailed values are in Table 3.

**Figure 2.**
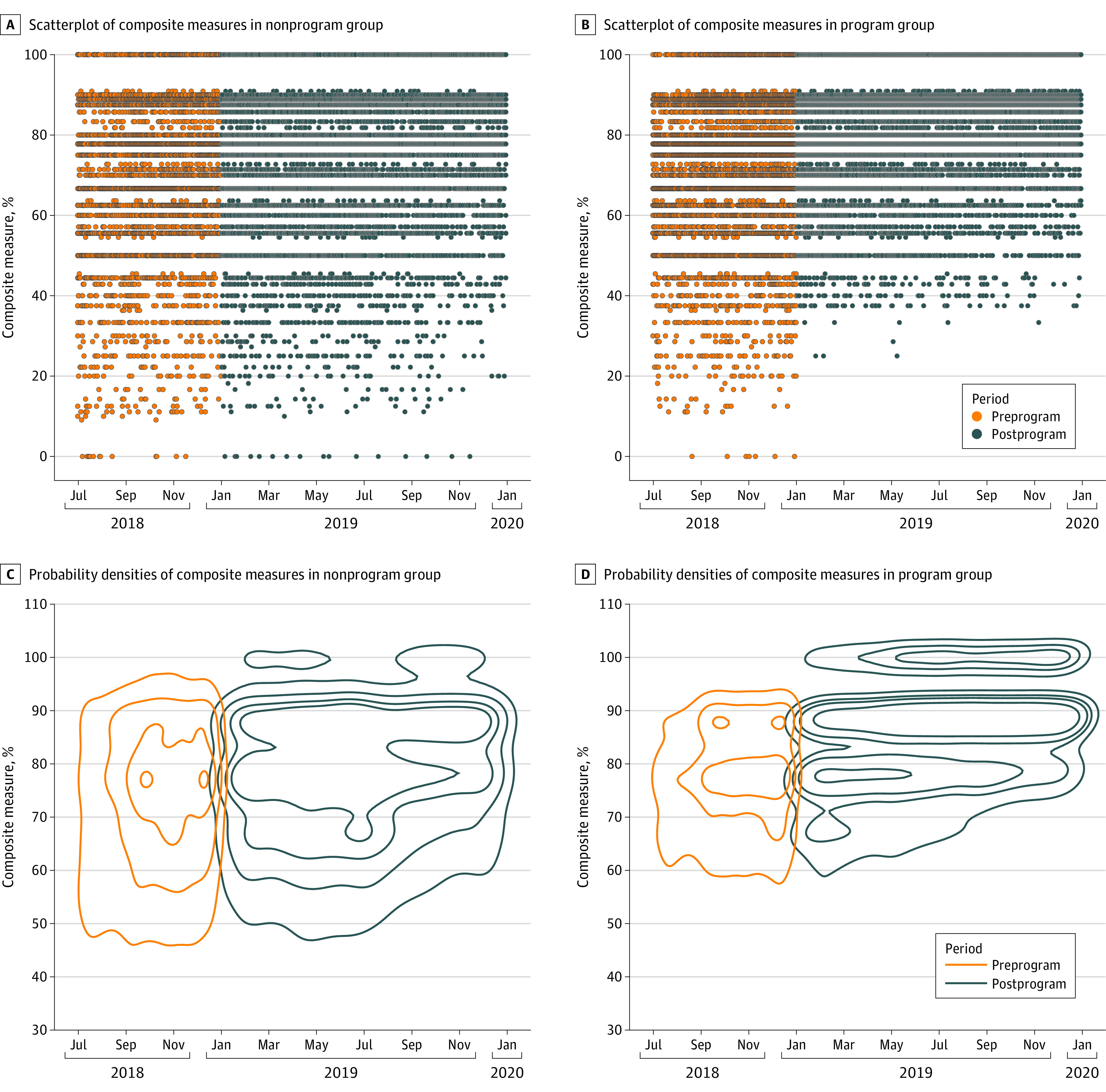
The Distribution of Composite Measure in the Program Group vs the Nonprogram Group A and B, Scatterplots of composite measures in the nonprogram group and program group during the preprogram and postprogram period, indicating that the majority of low scores disappeared postprogram in the program group. C and D, Probability densities of composite measure in the nonprogram group and program group during the preprogram and postprogram period, which was depicted with the time course. A block of 100% score appears postprogram in the program group. The orange color section represents the baseline, and the blue section depicts densities in the postprogram term.

The ITS model further determined the program’s outcomes on changes in the linear regression slope and level (Figure 1). The ITS result also showed that the program was associated with an increase in KPIs achieved per patient per week (slope change in short-term period, 0.36%; 95% CI, 0.20% - 0.52%; *P* < .001; level change in long-term period, 9.64%; 95% CI, 4.58% - 14.69%; *P* < .001), and in all-or-none success (slope change in short-term period, 0.34%; 95% CI, 0.23% - 0.46%; *P* < .001; level change in long-term period, 5.89%; 95% CI, 0.19% - 11.59%; *P* = .04) (Table 3). The result remained the same after removing intravenous rt-PA (eFigure 4 in Supplement 1). The program was not significantly associated with the level change in short-term period and the slope change in long-term period in both KPIs achieved per patient and all-or-none success. Daily composite score plots suggested that the program was associated with an immediate change by abolishing most of the low scores less than 40% (Figure 2A and 2B). Bivariate probability densities between composite measures and time points were analyzed, which produced distinctive blocks of probability densities before and after the program. A pronounced block consisting of density contour with 100% of composite measure was observed in the program group, which differs from that of the nonprogram group (Figure 2C and 2D).

**Table 3.** Comparison of Adherence to Evidence-Based KPIs After Program in Patients With Acute Ischemic Stroke Between the Program vs Nonprogram Group During Short-term and Long-term Periods in the Interrupted Time Series Model

Variable	Short-term period^a^				Long-term period^a^			
	Slope change, % (95% CI)^b^	*P* value	Level change, % (95% CI)^c^	*P* value	Slope change, % (95% CI)^b^	*P* value	Level change, % (95% CI)^c^	*P* value
Composite measure	0.36 (0.20 to 0.52)	<.001	0.65 (−1.64 to 2.94)	.58	−0.04 (−0.22 to 0.13)	.61	9.64 (4.58 to 14.69)	<.001
All-or-none score	0.34 (0.23 to 0.46)	<.001	−0.69 (−2.46 to 1.08)	.45	0.05 (−0.14 to 0.24)	.63	5.89 (0.19 to 11.59)	.04
KPIs at the beginning of hospitalization, %								
NIHSS assessment	0.65 (0.27 to 1.03)	.001	−0.12 (−5.77 to 5.54)	.97	0.01 (−0.42 to 0.45)	.96	17.59 (4.87 to 30.32)	.01
Intravenous rt-PA	0.24 (0.01 to 0.46)	.04	−2.01 (−5.35 to 1.33)	.24	−0.04 (−0.25 to 0.16)	.69	3.71 (−2.52 to 9.94)	.25
Early antithrombotics	0.26 (−0.02 to 0.55)	.08	0.43 (−3.78 to 4.64)	.84	−0.40 (−0.780 to −0.02)	.04	8.47 (−2.33 to 19.28)	.13
DVT prophylaxis	1.16 (0.22 to 2.11)	.02	−2.10 (−16.24 to 12.04)	.77	−0.02 (−1.04 to 0.99)	.97	36.45 (6.69 to 66.22)	.02
Dysphagia screening	0.92 (0.54 to 1.30)	<.001	2.48 (−3.06 to 8.01)	.38	0.53 (0.15 to 0.90)	.007	26.80 (16.08 to 37.52)	<.001
Rehabilitation evaluation	0.36 (−0.21 to 0.93)	.22	−3.99 (−12.03 to 4.06)	.33	−0.72 (−1.33 to −0.11)	.02	5.72 (−11.70 to 23.14)	.52
KPIs at discharge, %								
Antithrombotics	0 (−0.23 to 0.23)	.99	3.11 (−0.36 to 6.58)	.08	0.02 (−0.31 to 0.34)	.91	0.46 (−9.02 to 9.95)	.92
Antihypertensive medication	0.32 (−0.15 to 0.79)	.18	2.85 (−4.06 to 9.76)	.42	0.09 (−0.46 to 0.64)	.75	16.79 (0.64 to 32.95)	.04
Antidiabetic medication	0.54 (−0.03 to 1.11)	.07	0.95 (−7.60 to 9.50)	.83	0.30 (−0.23 to 0.83)	.27	10.68 (−5.05 to 26.41)	.19
Lipid-lowering for LDL-C >100 mg/dL^d^	0.19 (−0.12 to 0.50)	.24	1.76 (−2.77 to 6.29)	.45	−0.14 (−0.44 to 0.17)	.39	7.29 (−1.63 to 16.20)	.11
Anticoagulation for atrial fibrillation	1.03 (0.01 to 2.04)	.05	3.56 (−11.69 to 18.81)	.65	0.46 (−0.72 to 1.63)	.45	17.48 (−17.55 to 52.50)	.33
Smoking cessation	−0.05 (−0.16 to 0.07)	.42	1.05 (−0.68 to 2.79)	.24	−0.06 (−0.21 to 0.09)	.43	- 0.09 (−4.55 to 4.36)	.97
Clinical outcome at discharge, %								
Severe disability or death	−0.16 (−0.31 to −0.01)	.04	−2.10 (−4.36 to 0.16)	.07	−0.15 (−0.29 to −0.01)	.04	−5.18 (−9.49 to −0.86)	.02
Modified Rankin Scale score	−0.03 (−0.07 to 0.01)	.09	0.17 (−0.38 to 0.72)	.54	0 (−0.04 to 0.03)	.81	−0.37 (−1.47 to 0.73)	.51

Abbreviations: DVT, deep venous thrombosis; KPI, key performance indicator; LDL-C, low-density lipoprotein cholesterol; NIHSS, National Institutes of Health Stroke Scale; rt-PA, recombinant tissue-type plasminogen activator.

^a^ Short-term period: February 1, 2019, to July 31, 2019; long-term period: August 1, 2019, to January 31, 2020.

^b^ Data are for the slope change after program implementation in the program group in reference to the nonprogram group.

^c^ Data are for the level change after program implementation in the program group in reference to the nonprogram group.

^d^ To convert to millimoles per liter, multiply by 0.0259.

A reduced slope and level on the proportion of severe disability or death at discharge in the postprogram period was also noticed compared with the preprogram period. The slope change in the short-term period was −0.16% (95% CI, −0.31% to −0.01%; *P* = .04), and level change in the long-term period was −5.18% (95% CI, −9.49% to −0.86%; *P* = .02) (Table 3). The distribution of mRS score is shown in eFigure 5 in Supplement 1.

The outcomes of the program on individual KPIs were also analyzed. The results suggest that the program was statistically associated with an increased linear trend (slope) of NIHSS assessment, deep vein thrombosis (DVT) prophylaxis, dysphagia screening, and intravenous rt-PA in the short-term period, but not the remaining 8 individual KPIs. The program was also significantly associated with increased levels of NIHSS assessment, DVT prophylaxis, dysphagia screening, and antihypertensive medication at discharge in long-term period, but not the remaining 8 individual KPIs (Table 3). The DID model (Table 2) showed an even more pronounced statistical difference of the measures between the program and nonprogram group, demonstrating that the program was significantly associated with an increase in 10 out of 12 individual KPIs, except intravenous rt-PA and dysphagia screening.

Daily and monthly mean scores for all individual KPIs identified 3 KPIs still needing further improvement even after the program: rt-PA, DVT prophylaxis, and anticoagulation for atrial fibrillation (eFigure 6, eFigure 7, and eFigure 8 in Supplement 1). eFigure 9 in Supplement 1 shows only 1 ridge (peak) with a score less than 80% in most individual hospitals at baseline in both groups. Nevertheless, a peak of 100% and a peak of 90% appear in the postprogram period of the program group, which were not observed in the nonprogram group.

### Sensitivity Analyses

A sensitivity analysis, including patients in tertiary hospitals and secondary hospitals, respectively, was conducted between the program and nonprogram groups. The results suggest that the composite and all-or-none scores remained higher in the program group than those in the nonprogram group, both in short-term and long-term periods (eTable 5 and eTable 6 in Supplement 1).

## Discussion

This large general population quality improvement study on care programs among more than 45 000 patients with stroke used ITS and DID models for data analysis, demonstrating that the multilevel system program was associated with substantial increases in clinician adherence to stroke guidelines in routine clinical practice. In addition, the program was associated with an immediate change by abolishing low composite scores and increasing its score of 100%. Importantly, the program reduced the proportion of severely disabled or deceased patients with AIS at discharge.

On the basis of data reporting in a structured learning environment, the GWTG-Stroke program implementation achieved substantial improvements in the US. It has generated a global change in quality stroke care,^2^ as many developing and developed countries have adopted or adapted it to improve clinician adherence to stroke guidelines.^3,10,11,12,13,14^ Different from the GWTG-Stroke program, the CASE program was supported by an automated medical record data capture system and continuous feedback from a central quality initiative team via less expensive videoconferencing, and the results suggest that quality improvement programs are feasible in clinical practice and can still be successful in a less well-financed national health system. The automated medical record data capture system has been applied in China for a long time and might be generalized to other countries in different languages. Furthermore, this program could be applied in cardiovascular disease and the areas where health data integration and sharing are still far off, while the overcrowding of patients and heavy individual clinical workloads form the barriers for clinicians to implementing evidence-based care.

In the ITS model in this study, there was a secular trend in quality care improvement, which might be associated with China’s national initiatives for improving quality in stroke care. In addition, the request of documentation might play a reminder role for care practitioners to enhance adherence to guidelines in the nonprogram group and preprogram period of the program group. Moreover, practitioners might feel peer pressure to improve care quality if they are aware that this is a quality improvement program. Despite these possible confounders, the illuminating statistic visualizations show clear evidence that the program was associated with an immediate change, suggesting that the improving outcomes were not due to documentation itself, but due to the direct outcomes of the program.

The GOLDEN trial^3^ implemented a multifaceted quality improvement program to improve hospital personnel adherence to evidence-based performance measures in AIS and reported that it significantly improved the composite but not the all-or-none scores in China. Nevertheless, in this CASE program, the program was associated with substantial increases in both of them. Some possible reasons may explain the difference. First, the CASE program was a combination of multiple strategies, including regulation, supervision, training, and group problem-solving, which might generate larger effect sizes.^15^ Second, the video program was delivered monthly by a central quality care initiative team, which served as a repeated reminder for practitioners to condition their memories and behaviors. Third, the video conference included 3 to 4 hospitals together, which might generate a marketing effect to more efficiently change practitioners’ conduct for care quality.^16^

Of note, the care quality improvement in this study was associated with reduction in the proportion of severe disability or death (mRS 5-6) at discharge. Similar findings were reported that participation in GWTG-Stroke was associated with increases in discharge to home and reduced mortality at 30 days and 1 year, both of which are of great clinical importance.^17^ Increased adherence to in-hospital KPIs could help prevent early complications and deterrence of early infarct recurrence.^18,19^ The combination of all KPIs might generate a summation on improving neurological function, even in the short-term period after program, which lends support to wider implementation of such programs for hospitalized patients with stroke.

Intuitive evidence from the scatterplots of individual KPI scores extend the findings from the ITS and DID models, clearly identifying 3 important KPIs that still require further improvement: intravenous thrombolysis, DVT prophylaxis, and anticoagulation for atrial fibrillation at discharge (eFigure 7 and eFigure 8 in Supplement 1). These KPIs are particularly challenging in an Asian population, as in the current study, because of evidence-based concerns about higher bleeding risk in the Asian population among physicians and patients.^20^ In addition, DVT prophylaxis in China varied substantially among patients by age and stroke severity and among hospitals by location, bed number, and annual stroke discharges,^21^ which may explain its great fluctuation on the scatterplots. The underuse of rt-PA among patients with AIS, similar to the GOLDEN trial, prompts a stronger program on a national scale. The National Health Commission in China has listed “improving the rate of reperfusion treatment in AIS”^22^ as 1 of the national medical quality and safety improvement goals in 2021. The impact on patient care and outcomes will be of great interest.

### Limitations

Our study has several limitations. First, the absence of a randomized comparison group had the possibility of residual confounding and inherent selection bias. However, the baseline characteristics of the hospitals in the nonprogram group were matched with those in the program group. Second, the number of patients within the therapeutic time window of 4.5 hours for rt-PA administration was not available; thus, we could only calculate a relative proportion of rt-PA treatment among all patients with stroke admitted within 1 week of symptom onset. Third, the definition of performance measures in this study was described on the basis of the formula released by China Stroke Center Alliance, which is applicable only in China. Fourth, although we conducted propensity score weighting to match characteristics of both hospitals and patients between the program and nonprogram groups, clustering was not included in adjustment of DID analysis. Additionally, the current study did not include postdischarge outcomes. The long-term outcomes are still under collection for analysis.

## Conclusions

In this quality improvement study, a multilevel, centrally supported quality of care program was associated with substantial increases in clinician adherence to stroke guidelines and reduced the proportion of severely disabled or deceased patients with AIS at discharge in China. This quality program model with automated medical record data capture system and continuous feedback from a central quality care initiative team via video conference may serve as a model in a much broader range in the world for stroke care quality improvement, especially during the current COVID-19 pandemic.

## Notes

Supplement 1. **eAppendix.** eMethods **eTable 1.** The Designed Formula of KPIs **eTable 2.** Adherence to Evidence-Based KPIs in Patients with Acute Ischemic Stroke in the Program Group During 3 Periods **eTable 3.** Adherence to Evidence-Based KPIs in Patients with Acute Ischemic Stroke in the Nonprogram Group During 3 Periods **eTable 4.** Characteristics of Hospitals and Patients in Program and Nonprogram Group Before and After Propensity Score Weighting **eTable 5.** Adherence to Evidence-Based KPIs Between the Program and Nonprogram Group During Short-term and Long-term Period Among the Tertiary Hospitals for Sensitivity Analysis **eTable 6.** Adherence to Evidence-Based KPIs Between Program Group and Nonprogram Group During Short-term and Long-term Period Among the Secondary Hospitals for Sensitivity Analysis **eFigure 1.** The Automated Chart Data Capture System **eFigure 2.** Flowchart of Centrally-Supported Quality of Care Programs via Video Conference **eFigure 3.** Overview of Composite Measure, All-Or-None Scores, and Severe Disability or Death at Discharge Between the Program and Nonprogram Groups **eFigure 4.** The Segmented Regression Model and Statistical Results for the Composite Measures and All-or-None Scores After Removing Intravenous rt-PA in the Program and Nonprogram Groups **eFigure 5.** The Distribution of mRS Score at Discharge in the Program Group and Nonprogram Group During Preprogram Period and Postprogram Period **eFigure 6.** Individual KPIs Plotted With the Time Course in the Program vs Nonprogram Group **eFigure 7.** Scatterplots for Individual KPIs in the Program Group **eFigure 8.** Scatterplots for Individual KPIs in the Nonprogram Group **eFigure 9.** Ridge Plots Representing the Probability Density Distributions of Composite Measure

Supplement 2.
CASE Investigators
