# Supplementary material for: Evaluation of a Multilevel Program to Improve Clinician Adherence to Management Guidelines for Acute Ischemic Stroke
Source: JAMA Netw Open. 2022 May 6;5(5):e2210596. doi: 10.1001/jamanetworkopen.2022.10596 (PMC9077486; doi:10.1001/jamanetworkopen.2022.10596)
Supplement: Supplement 1. — eAppendix. eMethods eTable 1. The Designed Formula of KPIs eTable 2. Adherence to Evidence-Based KPIs in Patients with Acute Ischemic Stroke in the Program Group During 3 Periods eTable 3. Adherence to Evidence-Based KPIs in Patients with Acute Ischemic Stroke in the Nonprogram Group During 3 Periods eTable 4. Characteristics of Hospitals and Patients in Program and Nonprogram Group Before and After Propensity Score Weighting eTable 5. Adherence to Evidence-Based KPIs Between the Program and Nonprogram Group During Short-term and Long-term Period Among the Tertiary Hospitals for Sensitivity Analysis eTable 6. Adherence to Evidence-Based KPIs Between Program Group and Nonprogram Group During Short-term and Long-term Period Among the Secondary Hospitals for Sensitivity Analysis eFigure 1. The Automated Chart Data Capture System eFigure 2. Flowchart of Centrally-Supported Quality of Care Programs via Video Conference eFigure 3. Overview of Composite Measure, All-Or-None Scores, and Severe Disability or Death at Discharge Between the Program and Nonprogram Groups eFigure 4. The Segmented Regression Model and Statistical Results for the Composite Measures and All-or-None Scores After Removing Intravenous rt-PA in the Program and Nonprogram Groups eFigure 5. The Distribution of mRS Score at Discharge in the Program Group and Nonprogram Group During Preprogram Period and Postprogram Period eFigure 6. Individual KPIs Plotted With the Time Course in the Program vs Nonprogram Group eFigure 7. Scatterplots for Individual KPIs in the Program Group eFigure 8. Scatterplots for Individual KPIs in the Nonprogram Group eFigure 9. Ridge Plots Representing the Probability Density Distributions of Composite Measure [file jamanetwopen-e2210596-s001.pdf]

Supplemental Online Content

Chen Y, Gong X, Zhong W, et al; CASE Investigators. Evaluation of a multilevel program to improve clinician adherence to management guidelines for acute ischemic stroke. *JAMA Netw Open*. 2022;5(5):e2210596.
doi:10.1001/jamanetworkopen.2022.10596

eAppendix. eMethods
eTable 1. The Designed Formula of KPIs
eTable 2. Adherence to Evidence-Based KPIs in Patients with Acute Ischemic Stroke in the Program Group During 3 Periods
eTable 3. Adherence to Evidence-Based KPIs in Patients with Acute Ischemic Stroke in the Nonprogram Group During 3 Periods
eTable 4. Characteristics of Hospitals and Patients in Program and Nonprogram Group Before and After Propensity Score Weighting
eTable 5. Adherence to Evidence-Based KPIs Between the Program and Nonprogram Group During Short-term and Long-term Period Among the Tertiary Hospitals for Sensitivity Analysis
eTable 6. Adherence to Evidence-Based KPIs Between Program Group and Nonprogram Group During Short-term and Long-term Period Among the Secondary Hospitals for Sensitivity Analysis
eFigure 1. The Automated Chart Data Capture System
eFigure 2. Flowchart of Centrally-Supported Quality of Care Programs via Video Conference
eFigure 3. Overview of Composite Measure, All-Or-None Scores, and Severe Disability or Death at Discharge Between the Program and Nonprogram Groups
eFigure 4. The Segmented Regression Model and Statistical Results for the Composite Measures and All-or-None Scores After Removing Intravenous rt-PA in

© 2022 Chen Y et al. *JAMA Network Open.*

the Program and Nonprogram Groups
eFigure 5. The Distribution of mRS Score at Discharge in the Program Group and Nonprogram Group During Preprogram Period and Postprogram Period
eFigure 6. Individual KPIs Plotted With the Time Course in the Program vs Nonprogram Group
eFigure 7. Scatterplots for Individual KPIs in the Program Group
eFigure 8. Scatterplots for Individual KPIs in the Nonprogram Group
eFigure 9. Ridge Plots Representing the Probability Density Distributions of Composite Measure

This supplemental material has been provided by the authors to give readers additional information about their work.

© 2022 Chen Y et al. *JAMA Network Open.*

eAppendix: eMethods

Study Design

This CASE-Stroke study was a real world, non-randomized study. Only hospitals that had emergency departments, neurological wards and the capacity to administer intravenous thrombolysis were eligible for enrollment in our study. Hospitals were enrolled from a prospective multicenter stroke registry, Computer-based Online Database of Acute Stroke Patients for Stroke Management Quality Evaluation (CASE-II, NCT 04487340). Initiated in 2016, CASE-II was designed to examine the current status of stroke care in China in order to develop strategies to improve stroke care. The prespecified study period included two stages: the pre-intervention term - from August 1, 2018 to January 31, 2019, and the post-intervention term, from February 1, 2019 to January 31, 2020. Due to the consideration that the effects of interventions may take time to manifest, we divided post-intervention term into a short-term period from February 1, 2019 to July 31, 2019, and a long-term period from August 1, 2019 to January 31, 2020.

The clinical investigation was conducted according to the principle expressed in the Declaration of Helsinki. The Ethics Committee of the Second Affiliated Hospital, School of Medicine, Zhejiang University approved this study, and the protocol is registered on clinicaltrials.gov (NCT 03684629). Because patient information was de-identified and anonymized before being released to the researchers, the informed consent requirement was waived by the Institutional Review Board at the Second Affiliated Hospital of Zhejiang University.

Setting

An automated chart data capture system was used to reduce the enormous reporting burden from clinicians and ensure the authenticity and traceability of quality control data. It collected the patient data including demographic information, prior drug use, risk factors, treatment, laboratory information, severity evaluation and drug use during hospitalization and at discharge. The original medical documents from electronic or paper medical records were saved as images or portable document formats (PDF), then recognized, pre-processed, and sent to multiple Optical Character Recognition (OCR) engines to build documents with recognized text, which were subsequently re-segmented and synthesized in the post-processing. After the required patient data was extracted from the processed text, trained investigators checked their quality by comparing the original extraction sites where the system marked to verify. Three hundred medical documents were randomly selected to evaluate the error rate of the extraction, i.e., the rate of inconsistent data extracted by automated chart data capture system and manual input of neurologists, which was finally 3.4%. Key performance indicators (KPIs) are the objective quantitative

© 2022 Chen Y et al. *JAMA Network Open.*

management indicators that facilitate the hospital to follow. The KPI presentation system imported the verified data and calculated real-time KPIs according to the designed formula (**eTable 1**).

Data Collection and Quality Control

All medical documents from consecutive stroke patients admitted in stroke centers were collected according to the *International Classification of Diseases*, 10th revision (ICD-10) coding system (code I63 and G45). Data on patients during their hospitalization and at discharge were collected prospectively, and the local infrastructure and characteristics of each recruited hospital were also recorded. Range checks were used to check for out-of-range data and prompted the trained study investigators to correct or review the data collection outside of the predefined range. A data quality meeting was held monthly to review all of the hospital enrollment and extracted data records.

Hospitals and Participants

All hospital in CASE-II registry volunteered to participate in this study, then we conducted a prerandomization survey in all clusters and randomly select 30 hospitals to join the intervention group. Among the remaining hospitals, 28 hospitals were selected for the non-equivalent control (non-intervention) group after matching hospital characteristics by propensity score matching, who continued their routine care without receiving the intervention. Two hospitals in the non-intervention group refused to participate this study because they were unable to cooperate to participate in monthly video conferencing. Finally, we enrolled 30 hospitals in the intervention group and 28 hospitals in the non-intervention group. Patients were eligible if they were 18 years or older with AIS confirmed by brain computed tomography or magnetic resonance imaging within 7 days after symptom onset and admitted to wards directly or through the emergency department. Patients with other cerebrovascular diseases, such as cerebral hemorrhage and cerebral venous sinus thrombosis, were excluded.

Multilevel System Intervention

The program was an online, interactive, sustainable and modularized training program designed by a panel of stroke experts according to evidence-based clinical guidelines (**eFigure 2**). The program aimed to improve the care service for AIS patients and the medical record process at the individual, hospital and provincial levels. A professional medical central quality care initiative (QCI) team implemented the intervention at the hospitals through the following multiple strategies via monthly video conferencing:

1. A modularized standard template for medical records. According to the consensus statements and guidelines, the QCI team designed and deployed a modularized standard template for medical records to each

© 2022 Chen Y et al. *JAMA Network Open.*

participating hospital. By checking the boxes from the embedded template, clinicians integrated the evaluation plan and prescription plan involving the predefined performance measures into admission record and discharge record, respectively. According to the record, the corresponding care plan for each eligible stroke patient was then followed.

2. Centrally-supported continuing education. The QCI team required all neurologists from the intervention hospitals to attend the monthly video conference, aiming to educate them on the guideline recommendations and quality improvement related studies, and then increase their belief on the benefit of engaging them on the quality improvement process.

3. Continuous monitor-and-feedback. The QCI team used a real-time KPI presentation system and feedback reports on performance to encourage the hospital personnel to seek continuous improvement. The QCI team assigned an independent quality management account to the intervention group to log into the KPI presentation system to check their level of adherence to the predefined KPIs and compare them with the provincial average level. The QCI team required the clinicians to display the performance feedback reports as a slide for discussion on the monthly video conference.

4. Collaborative workshops. After the feedback report, the QCI team organized a collaborative workshop. The clinicians shared their problems and the possible causes, and the experts shared tools and ideas that had been developed, sought potential solutions and helped to tailor the implementations to improve the needs of each hospital. Then each hospital developed a simple improvement plan for the following month to ensure the implementation of all KPIs.

Outcomes

KPIs are the objective quantitative management indicators that facilitate the hospital to follow. The primary outcomes were the clinician adherence to the 12 predefined evidence-based KPIs, divided into two sections. Section one includes six indicators at the inpatient phase: 1. National Institutes of Health Stroke Scale (NIHSS) assessment, 2. Intravenous recombinant tissue-type plasminogen activator (rt-PA), 3. Early antithrombotics, 4. Deep venous thrombosis (DVT) prophylaxis, 5. Dysphagia screening, and 6. Rehabilitation evaluation. Section two includes six indicators at discharge: 7. Antithrombotics, 8. Antihypertensive medication, 9. Antidiabetic medication, 10. Lipid-lowering for Low-density lipoprotein cholesterol (LDL-C) >100 mg/dL, 11. Anticoagulation for atrial fibrillation, 12. Smoking cessation.

© 2022 Chen Y et al. *JAMA Network Open.*

Personnel adherence is expressed as a composite measure and an all-or-none score as co-primary outcomes based on the 12 KPIs. The composite measure is defined as the total number of eligible KPIs divided by total number of KPIs given to each eligible patient. The all-or-none score is defined as the proportion of eligible patients who received all of the KPIs. In addition, the percentage of each individual KPI was also calculated as the performed total number of patients divided by the total number of KPIs for eligible patients. The KPI of intravenous rt-PA was calculated as the total number of patients receiving rt-PA treatment divided by the total number of patients within 7 days of symptom onset, as the number of patients eligible for rt-PA treatment within the 4.5 h therapeutic time window was not available from participants.

The secondary outcomes were the distribution of modified Rankin Scale (mRS) score at discharge and the severe disability or death defined as mRS score 5-6 at discharge.

Statistical analysis

Baseline characteristics of hospitals and patients were compared between the intervention and non-intervention groups. Continuous variables were summarized as median (interquartile range), and categorical variables as frequency (percentage). The Wilcoxon rank-sum test was used for continuous variables and the Chi-square test for categorical variables. Means or medians for the weekly data of outcomes were calculated in the intervention and non-intervention groups.

The primary outcomes included the composite measures and the all-or-none scores, and the secondary outcome was the rate of severe disability or death defined as mRS score 5-6 at discharge. All individual KPIs were also analyzed.

Interrupted time series analysis (ITS), a longitudinal quasi-experimental approach, was used in the intervention group to evaluate the effect of the intervention program on the KPIs. To further control time-varying confounders which may affect both intervention and non-intervention groups, a controlled, interrupted time series analysis (CITS) was performed by recruiting data from the non-intervention group. To control for biases at the baseline level and trend, data from a defined pre-intervention period were included. Due to the consideration that the effects of interventions may take time to manifest, the post-intervention period was divided into short-term and long-term periods. The rapid change of rates in KPIs was observed in the short-term

© 2022 Chen Y et al. *JAMA Network Open.*

of the intervention and the gradual change in KPIs was observed over the long-term period, which were defined as slope change and level change, respectively. Segmented linear regression models were used to estimate the changes in levels and trends of rates after the implementation of the intervention. The regression model is as follows:

$$Y_t = \beta_0 + \beta_1 \times T + \beta_2 \times \text{Int} + \beta_3 \times \text{Post} + \beta_4 \times G + \beta_5 \times G \times T + \beta_6 \times G \times \text{Int} + \beta_7 \times G \times \text{Post} + \varepsilon,$$

Yt is the outcome variable at time *t* for the segmented linear regression model. *T* is a variable representing the time since the start of the study. *Int* (Intervention) is a binary variable indicating pre- (*Int*=0) or post- (*Int*=1) intervention period. *Post* is a continuous variable representing the time after the intervention and is coded 0 during the pre-intervention period. *G* represents the intervention (*G*=1) or non-intervention (G=0) group. *β0-β3* are parameter estimations for the non-intervention group, where *β0* represents the baseline level at the beginning of the time series, *β1* is the underlying pre-intervention trend, *β2* is the level change and *β3* is the slope change following the intervention. *β4-β7* represents the differences between the intervention and non-intervention group. *β4* is the difference in baseline levels; *β5*, the slope difference in the pre-intervention period; *β6*, the difference in level changes; and *β7*, the difference in slope changes. ε, random error. Cochrane-Orcutt autoregression would be used if we detected first-order autocorrelation in the data, and Durbin Watson statistics were close to the preferred value of 2.

In addition to the ITS model, difference-in-difference (DID) model was also used to analyze the differences in outcomes over the 2-time periods, pre-intervention and post-intervention. A standard DID regression model is as follow:

$$Y = \beta_1 \times \text{baseline} + \beta_2 \times \text{group} + \beta_3 \times (\text{baseline x group})$$

Y is the outcome of interest, i.e. composite or all-or-none scores, or individual KPI scores; baseline is a dummy variable for before or after intervention; and group is another dummy variable for intervention and non-intervention group. β_1, β_2, and β_3 are coefficients. β_3 is the target value that represents differential results of the interest outcomes between intervention and non-intervention group.

Nevertheless, a double robust regression model was used in this study, in which weights were added to balance potential unbalanced group distributions between intervention and non-intervention group, and additionally, those unbalanced characteristics of hospitals and patients as covariates were added. In brief, the multinomial propensity score weighting (mnps) function in the R twang package with propensity score weighting was used

to adjust group distribution balance. Both hospital and patient characters were used in the mnps function to extract weights. Then the svyglm regression function was used to examine the effect of intervention on the outcomes, which was adjusted using both hospital and patient characters as covariates.

In more detail for the DID analyses, composite and all-or-none scores, as well as all individual KPI scores (percentages) were calculated per day based on each hospital. To account for selection bias, a propensity score using the hospital and patient characteristics was created. We used the twang package in R to estimate the propensity scores and weighting of the comparison cases to estimate the average treatment effect on the treated (ATT). Multiple imputation (five times) was performed for missing values of all covariates by using the R package named mice. Only NIHSS and LDL-C had missing data at 10.2% and 3.8%, respectively. The data were divided into 4 groups: intervention group and non-intervention group during pre-intervention period and post-intervention period. According to these 4 groups, mnps (multinomial propensity scores) was calculated using characteristics of all hospital and patients by the R package named Twang, in which the parameter version was xgboost. The balance results were evaluated, and those characteristics of hospitals and patients (hospital grade, stroke unit, neurologist available at emergency department, number of stroke physicians, intravenous thrombolysis per year, annual stroke admission and hypertension) were not well balanced were selected again for the later DID regression model. Hospital characteristics include annual stroke admissions number, whether it is a teaching hospital, with stroke unit or not, neurologist available at emergency department, its grade being secondary or tertiary, bed numbers, neurological bed numbers, the capacity of performing endovascular therapy (EVT), stroke physicians number, intravenous thrombolysis treatment number, and EVT treatment number per year. Patient characteristics include age, sex, baseline NIHSS, LDL-C values, whether or not patients have hypertension, atrial fibrillation, diabetes, history of stroke, coronary heart disease, and smoking.

© 2022 Chen Y et al. *JAMA Network Open.*

eTable 1. The Designed Formula of KPIs

Key performance indicators.	Formula
**At the inpatient phase**	
NIHSS assessment	(Σ AIS patients who completed assessment of NIHSS /Σ the total number of AIS patients hospitalized in the same period) *100%
Intravenous rt-PA	(Σ AIS patients who received intravenous rt-PA thrombolytic therapy/Σ AIS patients who were admitted to hospital within 7 days of symptom onset in the same period)*100%
Early antithrombotics	(Σ AIS patients who received anti-thrombotics within 48 hours of admission /Σ the total number of AIS patients hospitalized in the same period) *100%
DVT prophylaxis	(Σ AIS patients who were unable to get out of bed within 48 hours of admission that were given DVT prophylactic measures including heparin and/or thrombus pump /Σ AIS patients who were unable to get out of bed within 48 hours of admission hospitalized in the same period) *100%
Dysphagia screening	(Σ AIS patients who had a history of swallow test /Σ the total number of AIS patients hospitalized in the same period) *100%
Rehabilitation evaluation	(Σ AIS patients who received rehabilitation evaluation /Σ the total number of AIS patients hospitalized in the same period) *100%
**At discharge**	
Antithrombotics	(Σ AIS patients who received anti-thrombotics at discharge/Σ the total number of AIS patients hospitalized in the same period) *100%
Antihypertensive medication	((Σ AIS patients with hypertension who were given anti-hypertensive medication at discharge /Σ the total

© 2022 Chen Y et al. *JAMA Network Open.*

	number of AIS patients with hypertension hospitalized in the same period) *100%
Antidiabetic medication	(Σ AIS patient with diabetes who were given hypoglycemic drugs at discharge/Σ the total number of AIS patients with diabetes hospitalized in the same period) *100%
Lipid-lowering for LDL-C >100 mg/dL	(Σ AIS patients with LDL-C >100 mg/dL, or treated with lipid lowering agent prior to admission, or LDL-C not documented who were given lipid lowering agent at discharge /Σ the total number of AIS patients with LDL-C >100 mg/dL, or treated with lipid lowering agent prior to admission, or LDL-C not documented hospitalized in the same period) *100%
Anticoagulation for atrial fibrillation	(Σ AIS patients with atrial fibrillation who received anticoagulants (e.g., heparin, low molecular heparin, warfarin, new oral anticoagulants) at discharge/Σ the total number of AIS patients with atrial fibrillation hospitalized in the same period) *100%
Smoking cessation	(Σ AIS patients who received smoking cessation counseling and/or health education during hospitalization/Σ the total number of AIS patients hospitalized in the same period) *100%

KPI=Key performance indicator; NIHSS=National Institutes of Health Stroke Scale; AIS=Acute ischemic stroke; rt-PA=Recombinant tissue-type plasminogen activator; DVT=Deep venous thrombosis; LDL=Low-density lipoprotein cholesterol.

© 2022 Chen Y et al. *JAMA Network Open.*

eTable 2. Adherence to Evidence-Based KPIs in Patients with Acute Ischemic Stroke in the Program Group During 3 Periods

	Baseline period[a]	Short-term period[b]	Long-term period[c]
	**No. of Events/Total Patients (%)**	**No. of Events/Total Patients (%)**	**No. of Events / Total Patients (%)**
Composite measure, mean±SD, %	73.2 ± 15.8	80.3 ± 12.6	85.4 ± 10.6
All-or-none score, n (%)	372 / 8001 (4.6)	1146 / 10901 (10.5)	1626 / 9819 (16.6)
**KPIs at the beginning of hospitalization, n (%)**			
NIHSS assessment	6757 / 8001 (84.5)	9726 / 10901(89.2)	9376 / 9819 (95.5)
Intravenous rt-PA	643 / 6755 (9.5)	976 / 9119 (10.7)	925 / 8157 (11.3)
Early antithrombotics	6281 / 6597 (95.2)	8859 / 9157(96.7)	8023 / 8204 (97.8)
DVT prophylaxis	615 / 1334 (46.1)	853 / 1666(51.2)	925 / 1466 (63.1)
Dysphagia screening	6646 / 8001 (83.1)	10162 / 10901(93.2)	9663 / 9819 (98.4)
Rehabilitation evaluation	3869 / 8001 (48.4)	8027 / 10901(73.6)	8673 / 9819 (88.3)
**KPIs at discharge, n (%)**			
Antithrombotics	6551 / 7257 (90.3)	9253 / 9742(95.0)	8204 / 8522(96.3)
Antihypertensive medication	3903 / 5967 (65.4)	5582 / 7940 (70.3)	5301 / 6561(80.8)
Antidiabetic medication	1549 / 2056 (75.3)	2279 / 2806 (81.2)	2179 / 2481 (87.8)
Lipid-lowering for LDL-C >100 mg/dL	5474 / 5959 (91.9)	7778 / 8208 (94.8)	6921 / 7190 (96.3)
Anticoagulation for atrial fibrillation	363 / 901 (40.3)	553 / 933(59.3)	583 / 777 (75.0)
Smoking cessation	7787 / 8001 (97.3)	10733 / 10901 (98.5)	9680 / 9819 (98.6)
**Clinical outcome at discharge**			
Severe disability or death, n (%)	696 / 8001 (8.7)	687 / 10901 (6.3)	609 / 9819 (6.2)
Modified Rankin Scale score, median (IQR)	1 (1,3)	1 (1,3)	1 (1,3)

KPI=Key performance indicator; NIHSS=National Institutes of Health Stroke Scale; rt-PA=Recombinant tissue-type plasminogen activator; DVT=Deep venous thrombosis; LDL-C=Low-density lipoprotein cholesterol.

[a] Pre-intervention period: August 1, 2018 to January 31, 2019; [b] Short-term period:February 1, 2019 to July, 31 2019;

© 2022 Chen Y et al. *JAMA Network Open.*

[c] Long-term period:August 1, 2019 to January 31, 2020.

© 2022 Chen Y et al. *JAMA Network Open.*

eTable 3. Adherence to Evidence-Based KPIs in Patients with Acute Ischemic Stroke in the Nonprogram Group During 3 Periods

	**Pre-intervention period[a]**	**Short-term period[b]**	**Long-term period[c]**
	**No. of Events/Total Patients (%)**	**No. of Events/Total Patients (%)**	**No. of Events / Total Patients (%)**
Composite measure, mean±SD , %	69.4 ± 18.8	72.6 ± 17.0	75.8 ± 15.8
All-or-none score, n (%)	266 / 4952 (5.4)	368 / 6320 (5.8)	400 / 5098 (7.8)
**KPIs at the beginning of hospitalization, n (%)**			
NIHSS assessment	3490 / 4952 (70.5)	4563 / 6320 (72.2)	3840 / 5098 (75.3)
Intravenous rt-PA	385 / 4150 (9.3)	530 / 5341 (9.9)	421 / 4278 (9.8)
Early antithrombotics	3986 / 4129 (96.5)	4834 / 5181 (93.3)	3975 / 4176 (95.2)
DVT prophylaxis	416 / 785 (53.0)	481 / 909 (52.9)	415 / 788 (52.7)
Dysphagia screening	3663 / 4952 (74.0)	5505 / 6320 (87.1)	4680 / 5098 (91.8)
Rehabilitation evaluation	2226 / 4952 (45.0)	3710 / 6320 (58.7)	3409 / 5098 (66.9)
**KPIs at discharge , n (%)**			
Antithrombotics	3867 / 4432 (87.3)	4840 / 5472 (88.5)	4014 / 4376 (91.7)
Antihypertensive medication	2255 / 3493 (64.6)	2759 / 4372 (63.1)	2178 / 3429(63.5)
Antidiabetic medication	933 / 1279 (72.9)	1154 / 1540 (74.9)	972 / 1227 (79.2)
Lipid-lowering for LDL-C>100 mg/dL	3224 / 3641 (88.5)	4081 / 4566 (89.4)	3404 / 3676 (92.6)
Anticoagulation for atrial fibrillation	255 / 576 (44.3)	306 / 608 (50.3)	285 / 470 (60.6)
Smoking cessation	4748 / 4952 (95.9)	6041 / 6320 (95.6)	4924 / 5098 (96.6)
**Clinical outcome at discharge**			
Severe disability or death, n (%)	204 / 4952 (4.1)	230 / 6320 (3.6)	203 / 5098 (4.0)
Modified Rankin Scale score, median (IQR)	1 (1,2)	1 (1,2)	1 (1,2)

Abbreviations: KPI, Key performance indicator; NIHSS, National Institutes of Health Stroke Scale; rt-PA,

© 2022 Chen Y et al. *JAMA Network Open.*

Recombinant tissue-type plasminogen activator; DVT, Deep venous thrombosis; LDL-C, Low-density lipoprotein cholesterol; IQR, Interquartile range.

[a] Pre-intervention period: August 1, 2018 to January 31, 2019; [b] Short-term period:February 1, 2019 to July, 31 2019; [c] Long-term period:August 1, 2019 to January 31, 2020

© 2022 Chen Y et al. *JAMA Network Open.*

eTable 4. Characteristics of Hospitals and Patients in Program and Nonprogram Group Before and After Propensity Score Weighting

	Intervention group					Non-intervention group							
	Pre-intervention period	Post-intervention period				Pre-intervention period				Post-intervention period			
	Reference	Unweighted	*P* value	weighted	*P* value	Unweighted	*P* value	weighted	*P* value	Unweighted	*P* value	weighted	*P* value
**Hospital characteristics**													
Stroke admissions per year, Mean (SD)	871.12 (429.8)	885.66 (430.4)	.09	870.90 (429.2)	.98	625.61 (487.8)	< .001	815.87 (603.4)	.002	612.36 (465.4)	< .001	816.07 (597.2)	< .001
Teaching hospital, Mean (SD), %	88.30 (32.1)	87.30 (33.4)	.11	88.0 (32.5)	.67	80.60 (39.6)	< .001	86.80 (33.8)	.11	80.30 (39.8)	< .001	86.60 (34.1)	.01
Stroke.unit, Mean (SD), %	87.40 (33.2)	87.40 (33.2)	.99	87.40 (33.2)	.99	78.30 (41.2)	< .001	90.90 (28.7)	< .001	77.80 (41.6)	< .001	90.10 (29.8)	< .001
Hospital grade, Mean (SD), %	82.10 (38.4)	80.90 (39.3)	.13	81.80 (38.6)	.67	65.0 (47.7)	< .001	76.60 (42.3)	< .001	64.40 (47.9)	< .001	74.90 (43.3)	< .001
Capacity of hospital department, Mean (SD)	1262.48 (656.9)	1243.05 (616.6)	.13	1257.60 (651.7)	.72	1131.29 (703.4)	< .001	1342.10 (859.3)	.002	1101.89 (683.3)	< .001	1338.27 (850.6)	< .001
Capacity of neurology department, Mean (SD)	73.27 (35.4)	72.52 (33.7)	.28	73.22 (35.1)	.95	62.88 (30.4)	< .001	72.17 (39.5)	.37	61.63 (29.6)	< .001	72.95 (39.4)	.75
Neurologist available at ED, Mean (SD), %	37.90 (48.5)	34.90 (47.7)	.002	37.90 (48.5)	.96	18.9 (39.2)	< .001	31.80 (46.6)	< .001	17.50 (38.0)	< .001	32.90 (47)	< .001

© 2022 Chen Y et al. *JAMA Network Open.*

Mechanical thrombectomy, Mean (SD), %	83.90 (36.7)	82.90 (37.6)	.16	83.80 (36.9)	.82	79.2 (40.6)	< .001	83.90 (36.8)	.93	79.60 (40.3)	< .001	83.90 (36.8)	.91
Number of stroke physicians, Mean (SD)	19.35 (11.8)	18.67 (10.9)	.003	19.21 (11.7)	.57	14.84 (10.5)	< .001	18.33 (13.1)	.01	14.19 (9.9)	< .001	18.49 (13)	.006
IVT treatment per year, Mean (SD)	140.02 (68.8)	140.77 (66.9)	.68	140.17 (68.4)	.98	98.71 (62.2)	< .001	126.47 (67.6)	< .001	94.97 (59.7)	< .001	127.52 (68.3)	< .001
EVT treatment per year, Mean (SD)	53.67 (54.3)	50.37 (52.3)	.002	53.39 (54.1)	.80	31.01 (48.3)	< .001	50.37 (60.4)	.08	27.03 (44)	< .001	54.23 (64.3)	.74
**Patient characteristics**													
Age, Mean (SD)	69.10 (10)	68.97 (9.5)	.52	69.09 (9.9)	.99	69.37 (10.6)	.31	68.90 (10)	.51	69.45 (10.5)	.10	69.15 (9.8)	.81
Female, Mean (SD), %	41.20 (39)	41.0 (37.1)	.86	41.30 (39)	.90	42.0 (41.7)	.46	41.50 (39.8)	.77	40.30 (40.7)	.28	40.60 (38.8)	.50
Baseline NIHSS, Mean (SD)	4.66 (4.2)	4.18 (3.8)	< .001	4.60 (4.2)	.53	4.40 (4.5)	.02	4.63 (4.5)	.85	4.41 (4.6)	.007	4.69 (4.5)	.74
LDL-C, Mean (SD), mg/dL	98.6 (27.1)	99.4 (27.1)	.12	98.6 (27.1)	.99	97.4 (0.8)	.08	97.4 (27.1)	.09	98.2 (27.1)	.32	98.2 (27.1)	.49
Hypertension, Mean (SD), %	66.30 (37.9)	65.70 (36.1)	.38	66.30 (37.9)	.98	64.40 (40.4)	.06	65.0 (38.4)	.25	63.20 (40.2)	< .001	63.60 (38.4)	.003
Atrial fibrillation, Mean (SD), %	7.60 (21.4)	6.70 (18.9)	.03	7.40 (20.9)	.59	8.50 (23.8)	.14	8.10 (22.0)	.42	7.80 (22.4)	.69	7.70 (21.2)	.81
Diabetes, Mean (SD), %	19.90 (31.8)	20.20 (30.3)	.70	20.20 (32)	.75	20.0 (34.2)	.93	19.70 (32.4)	.81	20.10 (33.1)	.88	20.50 (31.9)	.47
History of stroke/TIA, Mean (SD), %	23.20 (34)	22.50 (31.6)	.28	23.10 (33.8)	.83	23.10 (36.4)	.85	22.30 (34.4)	.35	23.40 (35.1)	.88	22.40 (33.1)	.28

© 2022 Chen Y et al. *JAMA Network Open.*

Coronary heart disease, Mean (SD), %	5.0 (17.6)	4.80 (16.4)	.65	5.0 (17.4)	.95	5.20 (18.9)	.70	4.50 (17.1)	.36	6.30 (20.4)	.002	5.30 (17.5)	.45
Smoking, Mean (SD), %	33.0 (37.6)	33.50 (35.8)	.51	33.10 (37.5)	.99	30.20 (38.6)	.004	31.40 (37.4)	.13	33.10 (39.4)	.98	33.50 (37.5)	.61

Abbreviations: CT, Computer tomography; EVT, Endovascular therapy; ED, Emergency department; TIA, Transient ischemic attacks; LDL-C, Low-density lipoprotein cholesterol; NIHSS, National Institute of Health Stroke Scale.

© 2022 Chen Y et al. *JAMA Network Open.*

eTable 5. Adherence to Evidence-Based KPIs Between the Program and Nonprogram Group During Short-term and Long-term Period Among the Tertiary Hospitals for Sensitivity Analysis

	Short-term period				Long-term period			
	**Slope change[a], % (95% CI)**	***P* value**	**Level change[b], % (95% CI)**	***P* value**	**Slope change[a], % (95% CI)**	***P* value**	**Level change[b], % (95% CI)**	***P* value**
Composite measure, mean±SD, %	0.28 (0.10,0.47)	< .001	-0.63 (-3.26,2.00)	.64	-0.15 (-0.38,0.09)	.23	6.64 (-0.13,13.41)	.06
All-or-none score, %	0.31 (0.11,0.50)	< .001	-1.55 (-4.49,1.38)	.30	-0.004 (-0.26,0.25)	.97	2.87 (-4.75,10.49)	.46
**KPIs at the beginning of hospitalization, %**								
NIHSS assessment,	0.31 (-0.13,0.76)	.18	-2.25 (-8.88,4.39)	.51	-0.19 (-0.74,0.35)	.49	5.71 (-10.25,21.67)	.49
Intravenous rt-PA	0.23 (-0.02,0.49)	.07	-3.64 (-7.44,0.16)	.06	-0.15 (-0.40,0.10)	.24	0.73 (-6.75,8.21)	.85
Early antithrombotics	0.36 (-0.04,0.76)	.08	0.61 (-5.19,6.41)	.84	-0.54 (-1.05,-0.02)	.04	12.67 (-1.69,27.03)	.09
DVT prophylaxis	0.71 (-0.30,1.72)	.17	-3.79 (-19.00,11.43)	.63	-0.06 (-1.20,1.08)	.91	23.54 (-10.45,57.54)	.18
Dysphagia screening	0.71 (0.22,1.20)	.005	0.77 (-6.20,7.74)	.83	0.25 (-0.22,0.71)	.30	19.92(6.68,33.15)	.004
Rehabilitation evaluation	0.30 (-0.27,0.86)	.31	-7.55 (-15.64,0.53)	.07	-1.01 (-1.77,-0.26)	.01	1.38 (-19.27,22.03)	.90
**KPIs at discharge, %**								
Antithrombotics	-0.01 (-0.26,0.25)	.97	2.73 (-1.08,6.53)	.16	0.07 (-0.32,0.46)	.73	-0.22 (-11.58,11.14)	.97
Antihypertensive medication	0.18 (-0.31,0.67)	.47	3.78 (-3.48,11.03)	.31	0.18 (-0.32,0.68)	.49	12.88 (-2.08,27.85)	.10
Antidiabetic medication	0.64 (-0.01,1.28)	.06	-3.56 (-13.23,6.10)	.47	0.32 (-0.29,0.94)	.30	6.49 (-11.79,24.77)	.49

© 2022 Chen Y et al. *JAMA Network Open.*

Lipid-lowering for LDL-C >100 mg/dL	0.25 (-0.02,0.52)	.08	0.84 (-3.26,4.94)	.69	-0.14 (-0.41,0.12)	.29	6.52 (-1.29,14.33)	.11
Anticoagulation for atrial fibrillation	0.73 (-0.36,1.82)	.20	3.83 (-12.53,20.19)	.65	0.73 (-0.62,2.08)	.29	6.72 (-33.43,46.88)	.74
Smoking cessation	-0.07 (-0.21,0.07)	.33	0.73 (-1.37,2.84)	.50	-0.07 (-0.22,0.08)	.35	-0.90 (-5.25,3.46)	.69

KPI=Key performance indicator; NIHSS=National Institutes of Health Stroke Scale; rt-PA=Recombinant tissue-type plasminogen activator; DVT=Deep venous thrombosis; LDL-C=Low-density lipoprotein cholesterol.

Short-term period: February 1, 2019 to July 31, 2019; Long-term period: August 1, 2019 to January 31, 2020.

[a] the slope change after the intervention implementation in the intervention group in reference to the non-intervention group.

[b] the level change after the intervention implementation in the intervention group in reference to the non-intervention group.

© 2022 Chen Y et al. *JAMA Network Open.*

eTable 6. Adherence to Evidence-Based KPIs Between Program Group and Nonprogram Group During Short-term and Long-term Period Among the Secondary Hospitals for Sensitivity Analysis

	Short-term period				Long-term period			
	**Slope change[a], % (95% CI)**	***P* value**	**Level change[b], % (95% CI)**	***P* value**	**Slope change[a], % (95% CI)**	***P* value**	**Level change[b], % (95% CI)**	***P* value**
Composite measure, mean±SD, %	0.56 (0.26,0.85)	< .001	6.23 (1.85,10.60)	.006	0.24 (-0.06,0.54)	.11	22.47 (13.55,31.38)	< .010
All-or-none score, %	0.45 (0.18,0.73)	< .001	2.08 (-2.04,6.19)	.33	0.17 (-0.18,0.51)	.35	14.87 (4.56,25.17)	.006
**KPIs at the beginning of hospitalization, %**								
NIHSS assessment	1.66 (0.96,2.36)	< .001	6.89 (-3.61,17.39)	.20	0.75 (0.15,1.35)	.02	52.51 (34.66,70.35)	< .001
Intravenous rt-PA	0.32 (-0.10,0.74)	.14	1.71 (-4.59,8.01)	.60	0.19 (-0.26,0.63)	.41	11.77 (-1.57,25.11)	.09
Early antithrombotics	0.22 (-0.17,0.61)	.27	-0.48 (-6.31,5.34)	.87	0.02 (-0.24,0.28)	.88	1.44 (-6.50,9.38)	.72
DVT prophylaxis	2.58 (0.38,4.78)	.02	14.57 (-18.33,47.48)	.39	-1.02 (-3.48,1.44)	.42	93.09 (19.71,166.48)	.02
Dysphagia screening	1.68 (0.91,2.44)	< .001	9.58 (-1.76,20.92)	.10	1.47 (0.88,2.06)	< .001	52.87(35.34,70.39)	< .001
Rehabilitation evaluation	0 (-1.24,1.24)	.99	13.47 (-4.53,31.47)	.15	0.08 (-1.15,1.31)	.89	27.44 (-8.02,62.89)	.13
**KPIs at discharge, %**								
Antithrombotics	-0.01 (-0.50,0.48)	.98	5.12 (-2.27,12.52)	.18	-0.15 (-0.74,0.43)	.61	1.90 (-15.28,19.08)	.83
Antihypertensive medication	0.66 (-0.35,1.68)	.20	3.11 (-12.13,18.34)	.69	-0.40 (-1.45,0.64)	.45	28.91 (-2.25,60.06)	.07
Antidiabetic medication	-0.14 (-1.50,1.23)	.85	14.16 (-6.39,34.72)	.18	-0.23 (-1.60,1.14)	.74	18.21 (-22.62,59.04)	.38

© 2022 Chen Y et al. *JAMA Network Open.*

Lipid-lowering for LDL-C >100 mg/dL	0.06 (-0.51,0.63)	.83	6.43 (-2.08,14.94)	.14	-0.16 (-0.78,0.46)	.62	11.37 (-6.87,29.61)	.23
Anticoagulation for atrial fibrillation	1.38 (-0.94,3.70)	.25	7.91 (-26.23,42.04)	.65	-0.95 (-3.36,1.46)	.44	57.35 (-14.34,129.04)	.12
Smoking cessation	-0.06 (-0.40,0.27)	.72	2.36 (-2.68,7.40)	.36	-0.03 (-0.42,0.36)	.88	-1.80 (-13.26,9.67)	.76

KPI=Key performance indicator; NIHSS=National Institutes of Health Stroke Scale; rt-PA=Recombinant tissue-type plasminogen activator; DVT=Deep venous thrombosis; LDL-C=Low-density lipoprotein cholesterol.

Short-term period: February 1, 2019 to July 31, 2019; Long-term period: August 1, 2019 to January 31, 2020.

[a] the slope change after the intervention implementation in the intervention group in reference to the non-intervention group.

[b] the level change after the intervention implementation in the intervention group in reference to the non-intervention group.

© 2022 Chen Y et al. *JAMA Network Open.*

eFigure 1. The Automated Chart Data Capture System

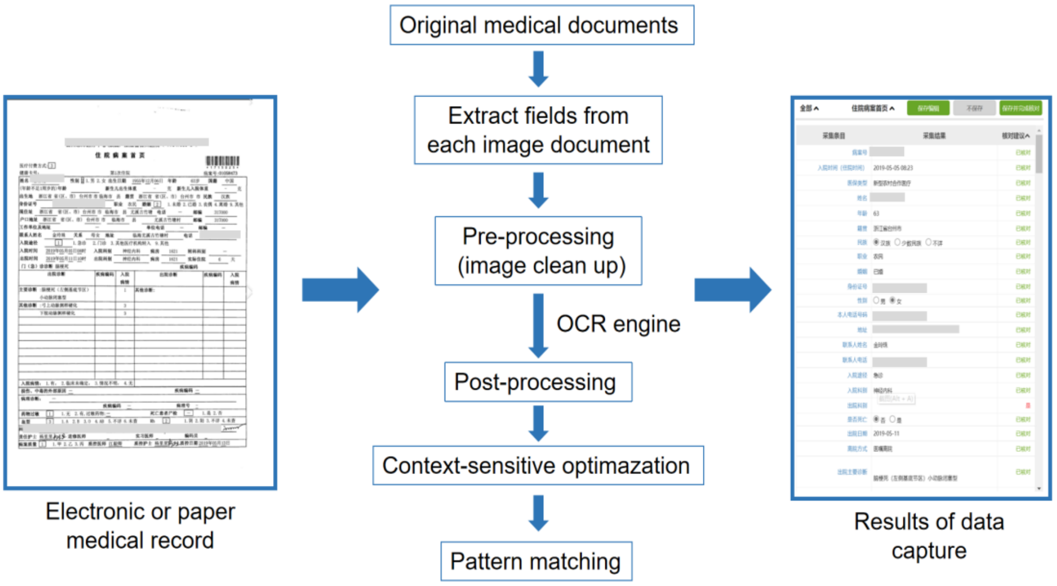

The original medical documents (left) were saved as portable document formats or images to build documents with recognized text (right) after data capture (middle, the optical character recognition (OCR) system pipeline).

© 2022 Chen Y et al. *JAMA Network Open.*

eFigure 2. Flowchart of Centrally-Supported Quality of Care Programs via Video Conference

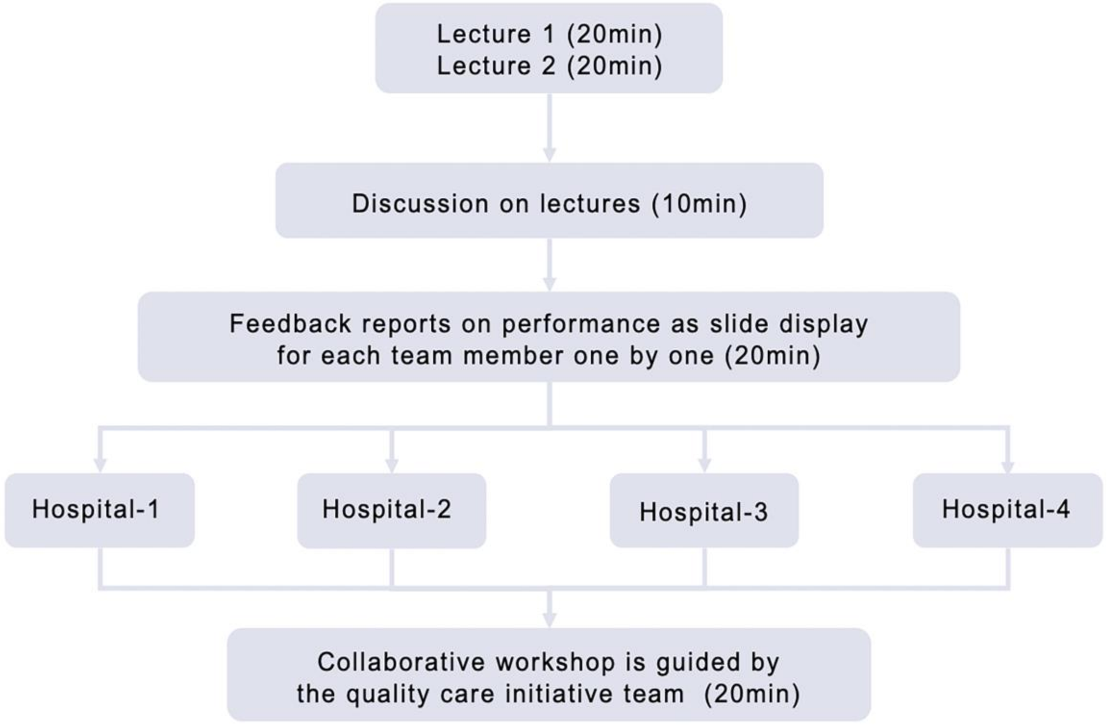

© 2022 Chen Y et al. *JAMA Network Open.*

eFigure 3. Overview of Composite Measure, All-or-None Scores, and Severe Disability or Death at Discharge Between the Program and Nonprogram Groups

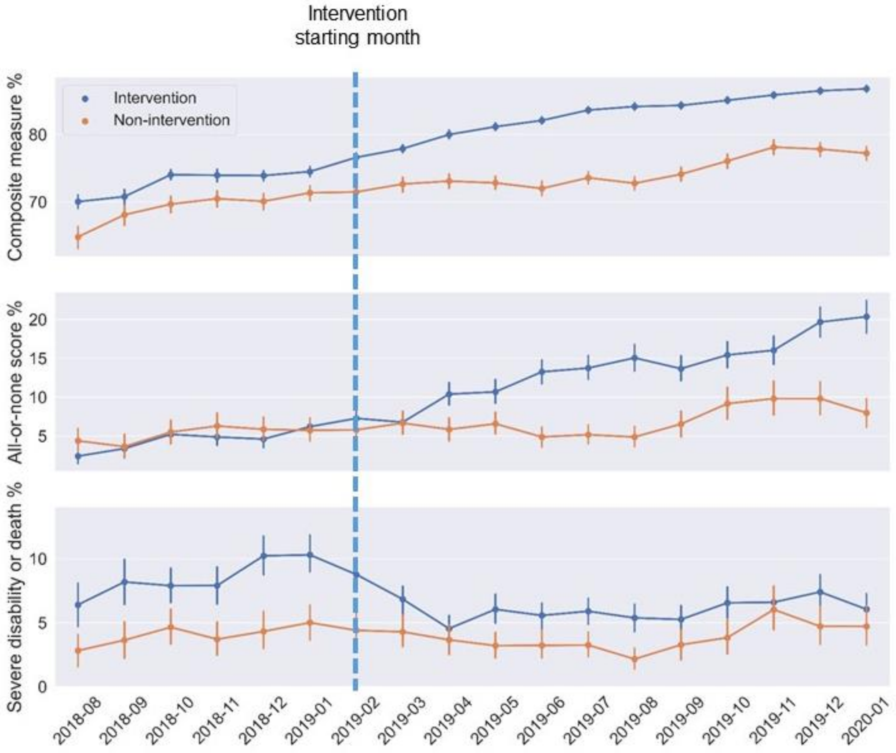

Average scores for each month were computed and plotted with 95% confidence interval as shown by a bar at each time point.

© 2022 Chen Y et al. *JAMA Network Open.*

eFigure 4. The Segmented Regression Model and Statistical Results for the Composite Measures and All-or-None Scores After Removing Intravenous rt-PA in the Program and Nonprogram Groups

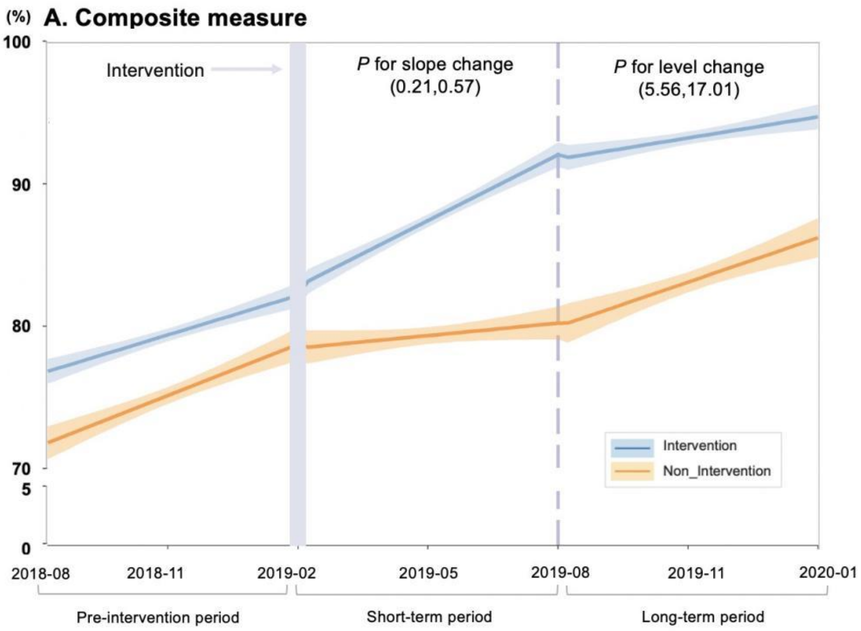

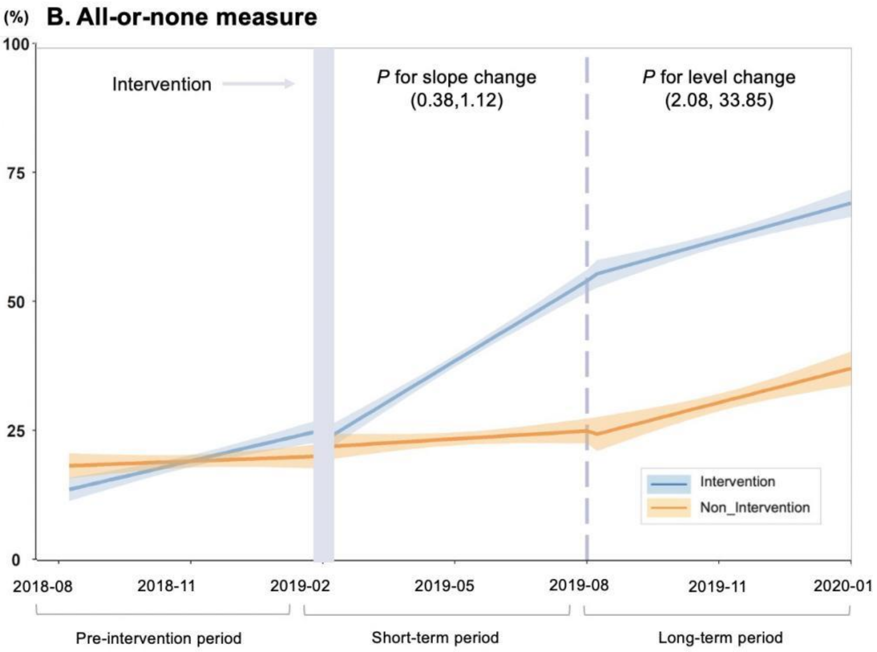

The model divided the time course into three periods: the pre-intervention, short-term and long-term post-intervention periods. Shaded regions indicate 95% confidence intervals; dashed lines represent divisions between short-term period and long-term period. **A**. The model shows the difference between the change in regression line slopes and levels between the intervention and non-intervention groups for the composite measure. **B**. The model indicates the difference between the changes in slopes and levels in the two groups for the all-or-none scores.

© 2022 Chen Y et al. *JAMA Network Open.*

eFigure 5. The Distribution of mRS Score at Discharge in the Program Group and Nonprogram Group During Preprogram Period and Postprogram Period

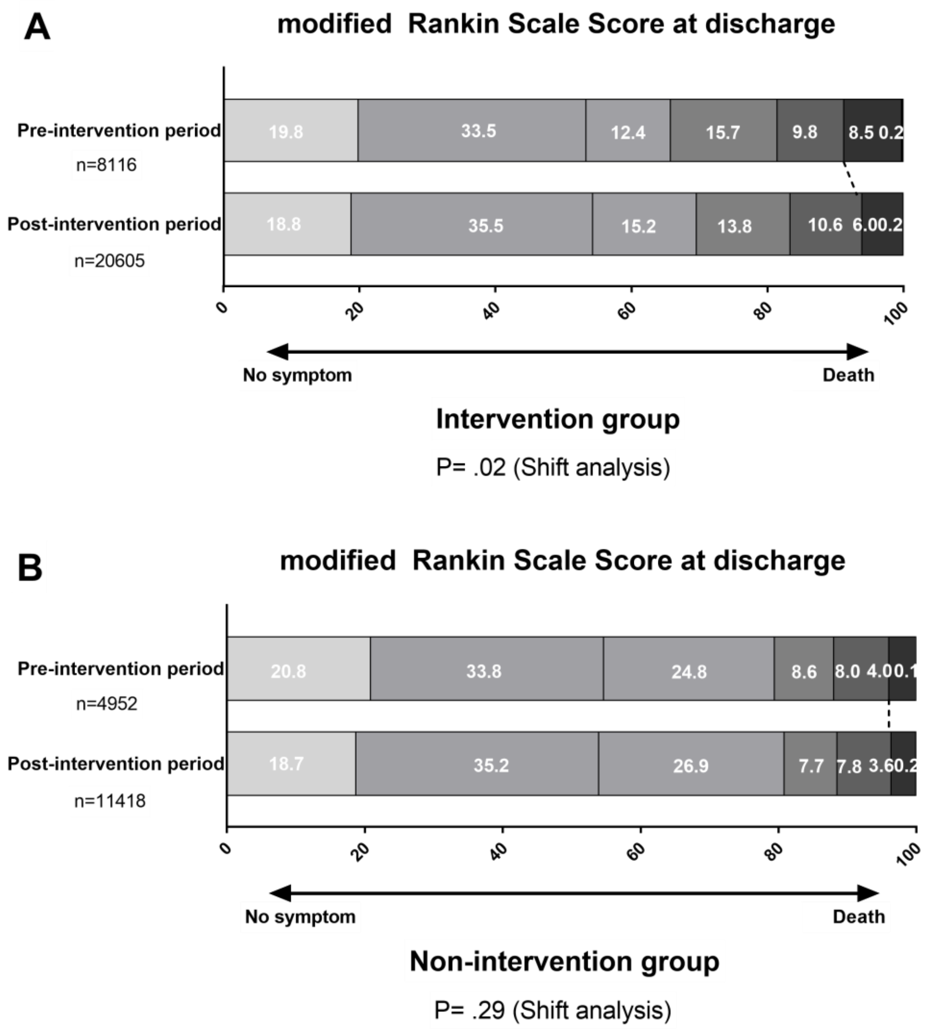

© 2022 Chen Y et al. *JAMA Network Open.*

eFigure 6. Individual KPIs Plotted With the Time Course in the Program vs Nonprogram Group

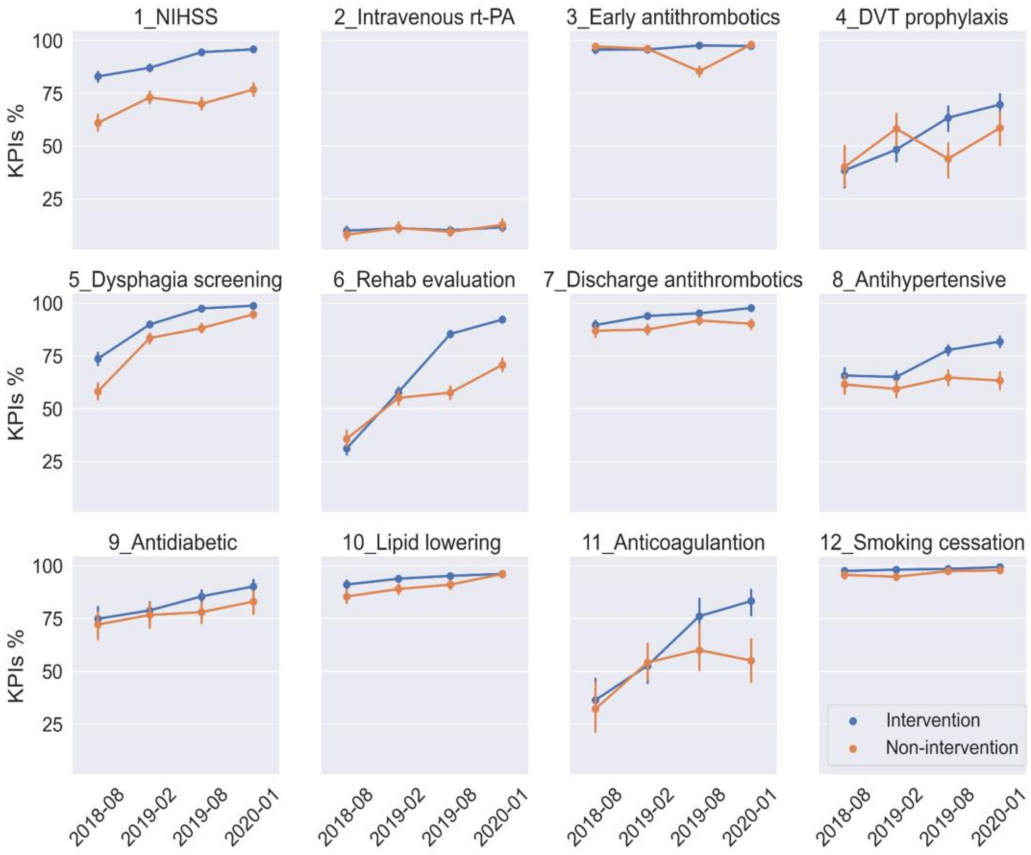

Average monthly composite measure and their 95% confidence intervals for four key months were selected and plotted with the time course. The first time point is the first month when the program was initiated; the second time point represents the 7th month, which is also the first month of the post-intervention short term; the third point is the 13th month, which is also the first month for the long term period; and the last point refers to the last month of the program.

© 2022 Chen Y et al. *JAMA Network Open.*

eFigure 7. Scatter Plots for Individual KPIs in the Program Group

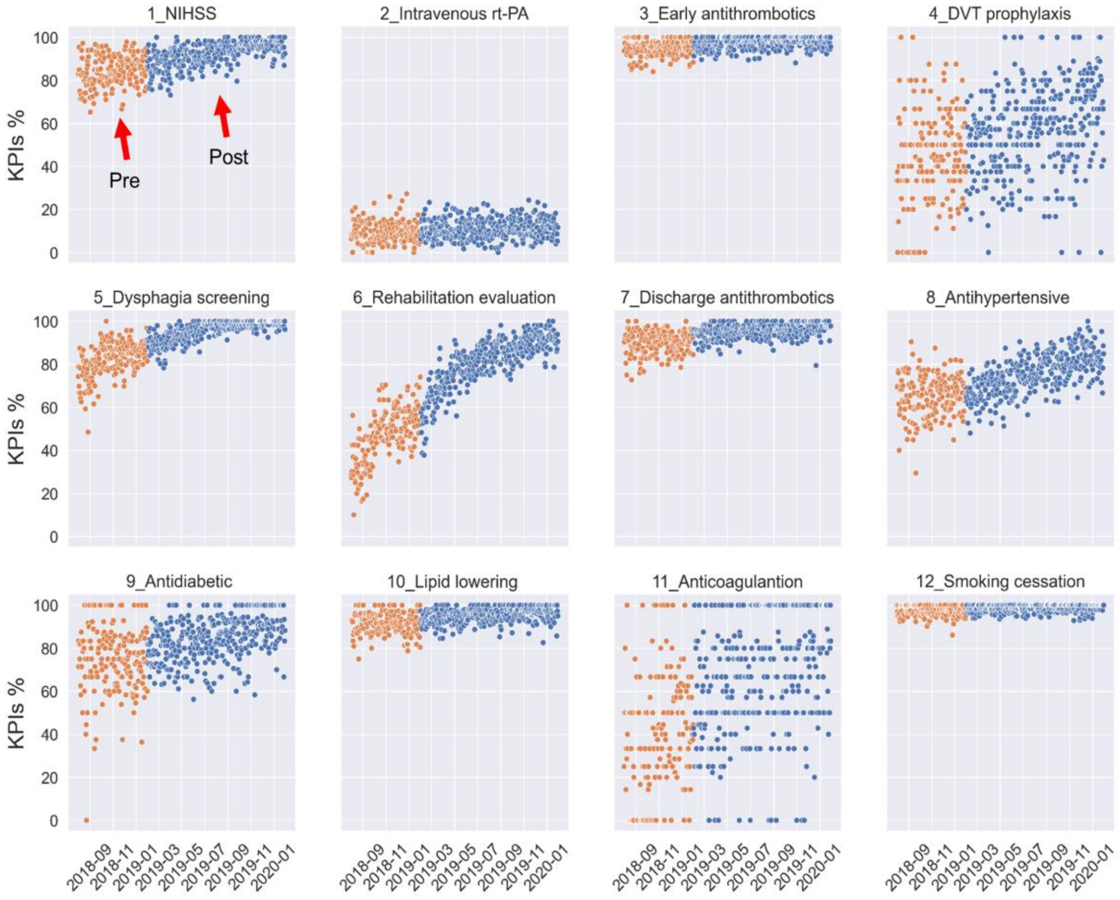

Average daily individual KPI scores were calculated and portrayed with the time course. The orange part represents the baseline (pre), while the blue part represents the post-intervention (post) period.

© 2022 Chen Y et al. *JAMA Network Open.*

eFigure 8. Scatter Plots for Individual KPIs in the Nonprogram Group

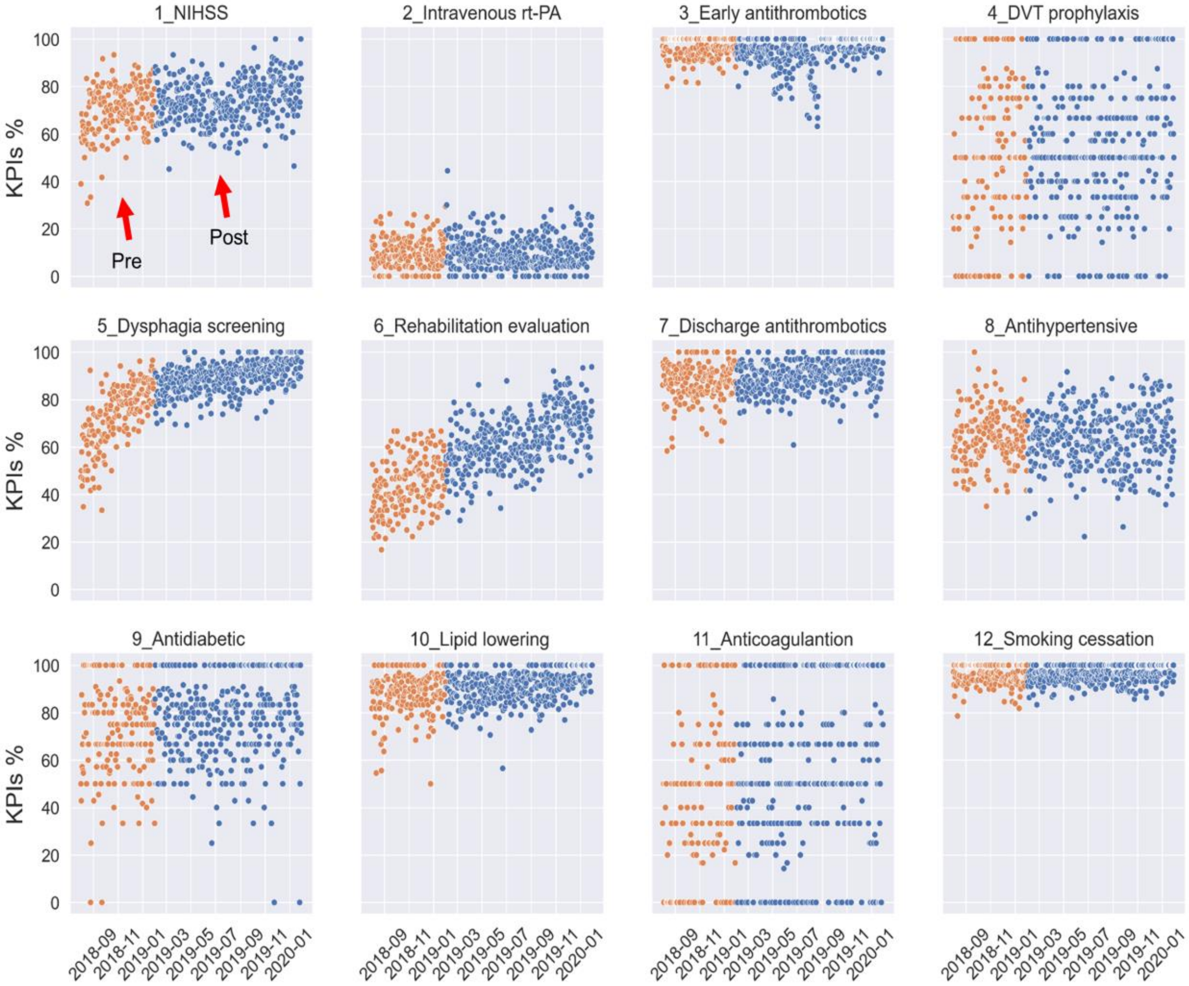

Average daily individual KPI scores were calculated and portrayed with the time course. The orange part represents the baseline, while the blue part represents the post-intervention period.

© 2022 Chen Y et al. *JAMA Network Open.*

eFigure 9. Ridge Plots Representing the Probability Density Distributions of Composite Measure

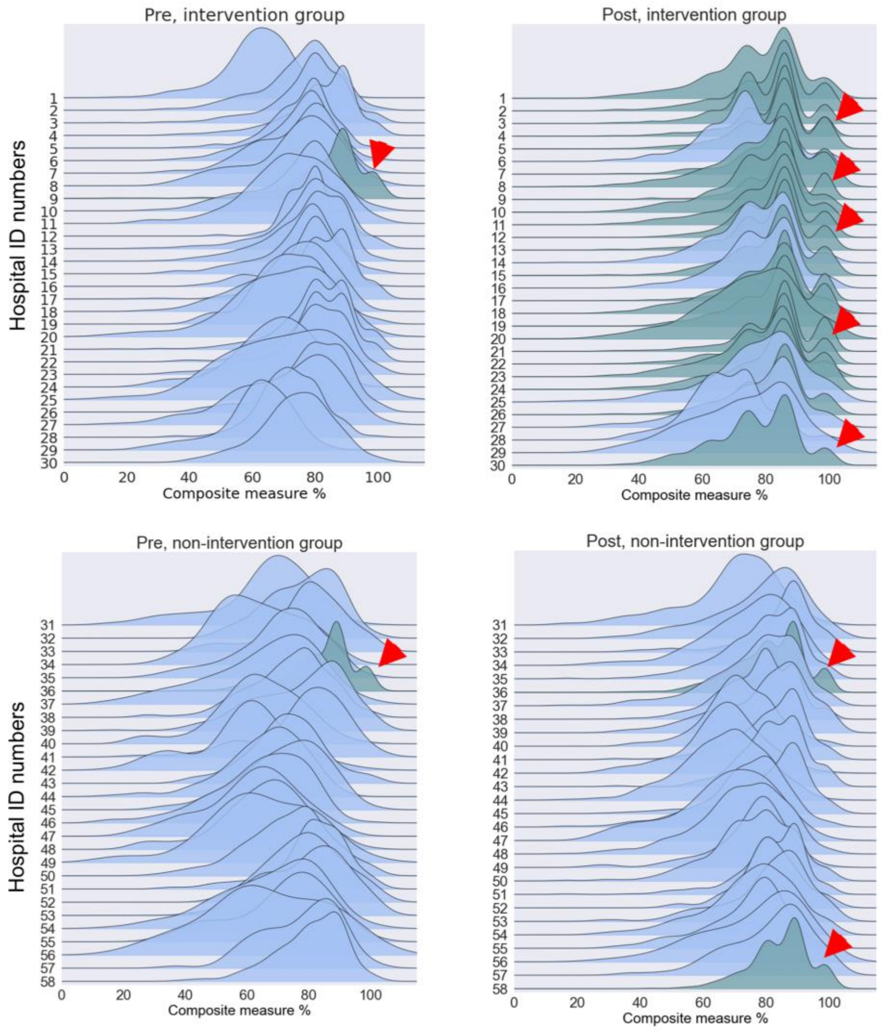

On the Y axis are hospital's ID numbers. A single ridge plot represents the distribution of composite measure for each hospital. The top row is the intervention group and the bottom is the non-intervention group. The left column represents the baseline score densities of the two groups, and the right are the score distribution in the post-intervention period. Red arrow heads are used to indicate the peak of 100% score in green ridges. The blue ridges represent the score distribution without the peak of 100% scores.

© 2022 Chen Y et al. *JAMA Network Open.*
