# Supplementary material for: Evaluation of a Multilevel Program to Improve Clinician Adherence to Management Guidelines for Acute Ischemic Stroke
Source: JAMA Netw Open. 2022 May 6;5(5):e2210596. doi: 10.1001/jamanetworkopen.2022.10596 (PMC9077486; doi:10.1001/jamanetworkopen.2022.10596)
Supplement: Supplement 2. — CASE Investigators [file jamanetwopen-e2210596-s002.pdf]

*Group Name(s): CASE Investigators							
***First Name and Middle Initial(s)**	***Last Name**	***Suffix (eg, Jr, III)**	Academic Degrees	Institution	Location (city, state/province, country)	Role or Contribution, eg, chair, principal investigator	Group (if more than 1 Group listed in the byline) and/or Subgroup (eg, Steering Committee)
Dongjuan	Xu		MD	Dongyang People's Hospital	Jinhua, Zhejiang, China	participated in implementing the study	
Chenglong	Wu		MD	Shaoxing People's Hospital	Shaoxing, Zhejiang, China	participated in implementing the study	
Chaochan	Cheng		MD	Yongkang First People's Hospital	Jinhua, Zhejiang, China	participated in implementing the study	
Haifang	Hu		MD	The First People's Hospital of Xiaoshan District	Hangzhou, Zhejiang, China	participated in implementing the study	
Xinzheng	Fu		MD	Haining People's Hospital	Jiaxing, Zhejiang, China	participated in implementing the study	
Xueli	Cai		MD	Lishui City Central Hospital and the Fifth Affiliated Hospital of Wenzhou Medical University	Lishui, Zhejiang, China	participated in implementing the study	
Weiguo	Tang		MD	Zhoushan Hospital, Wenzhou Medical University	Zhoushan, Zhejiang, China	participated in implementing the study	
Guomin	Xie		MD	Lihuili Hospital of Ningbo Medical Center	Ningbo, Zhejiang, China	participated in implementing the study	
Faming	Wang		MD	Tiantai People's Hospital	Taizhou, Zhejiang, China	participated in implementing the study	
Wei	Dong		MD	Yuyao People's Hospital	Ningbo, Zhejiang, China	participated in implementing the study	
Wenping	Gong		MD	Shengzhou People's Hospital, Shengzhou Branch Hospital of The First Affiliated Hospital of Zhejiang University	Shaoxing, Zhejiang, China	participated in implementing the study	
Weiqing	Chen		MD	Xianju County People's Hospital	Taizhou, Zhejiang, China	participated in implementing the study	
Xiaodong	Ma		MD	Haiyan People’s Hospital	Jiaxing, Zhejiang, China	participated in implementing the study	
Xiaoqing	Wu		MD	Xinchang County People's Hospital	Shaoxing, Zhejiang, China	participated in implementing the study	
Jianhua	Cheng		MD	The first affiliated hospital of Wenzhou Medical University	Wenzhou, Zhejiang, China	participated in implementing the study	
Yi	Wu		MD	Yiwu Central Hospital of Wenzhou Medical University	Jinhua, Zhejiang, China	participated in implementing the study	
Jiayong	Dai		MD	Linhai Second People's Hospital	Taizhou, Zhejiang, China	participated in implementing the study	
Hongxiang	Wang		MD	The First People's Hospital of Fuyang	Hangzhou, Zhejiang, China	participated in implementing the study	
Donggan	Jin		MD	Pujiang People's Hospital	Jinhua, Zhejiang, China	participated in implementing the study	
Fei	Wang		MD	The First People's Hospital of Jiashan County	Jiaxing, Zhejiang, China	participated in implementing the study	
Ai'ju	Wang		MD	Ningbo Fourth Hospital	Ningbo, Zhejiang, China	participated in implementing the study	
Yaqiong	Zhou		MD	People's Hospital of Panan County	Jinhua, Zhejiang, China	participated in implementing the study	
Junfang	Kang		MD	Fenghua District People's Hospital	Ning bo, Zhejiang, China	participated in implementing the study	
Gongchun	Huang		MD	Pinghu First People's Hospital	Jiaxing, Zhejiang, China	participated in implementing the study	
Ningyuan	Zhang		MD	Tongxiang First People's Hospital	Jiaxing, Zhejiang, China	participated in implementing the study	
Huadong	Huang		MD	Changxing County People's Hospital	Huzhou, Zhejiang, China	participated in implementing the study	
Guoping	Fu		MD	Shaoxing Second Hospital	Shaoxing, Zhejiang, China	participated in implementing the study	
Yong	Chen		MD	Ningbo Medical Center, Li Huili Eastern Hospital, .	Ningbo, Zhejiang, China	participated in implementing the study	
Yanbin	Zhang		MD	The First People's Hospital of Yuhang District	Hangzhou, Zhejiang, China	participated in implementing the study	
Qun	Gu		MD	Huzhou First People's Hospital	Huzhou, Zhejiang, China	participated in implementing the study	
Hongling	He		MD	Xinchang County Hospital of Traditional Chinese Medicine	Shaoxing, Zhejiang, China	participated in implementing the study	
Jun	Xu		MD	Ningbo Yinzhou District Second Hospital	Ningbo, Zhejiang, China	participated in implementing the study	

***First Name and Middle Initial(s)**	***Last Name**	***Suffix (eg, Jr, III)**	Academic Degrees	Institution	Location (city, state/province, country)	Role or Contribution, eg, chair, principal investigator	Group (if more than 1 Group listed in the byline) and/or Subgroup (eg, Steering Committee)
Kun	Han		MD	Hwa Mei Hospital, University of Chinese Academy of Sciences	Ningbo, Zhejiang, China	participated in implementing the study	
Risheng	Wu		MD	Yuhuan People's Hospital	Taizhou, Zhejiang, China	participated in implementing the study	
Kai	Fang		MD	Zhejiang Xiaoshan Hospital	Hangzhou, Zhejiang, China	participated in implementing the study	
Zhijun	Zheng		MD	Hengdian Wenrong Hospital	Jinhua, Zhejiang, China	participated in implementing the study	
Xiaoli	Shao		MD	Chun'an First People's Hospital (Zhejiang Provincial People's Hospital Chun'an Branch)	Hangzhou, Zhejiang, China	participated in implementing the study	
Weiqin	Ma		MD	Tongxiang Hospital of Traditional Chinese Medicine	Jiaxing, Zhejiang, China	participated in implementing the study	
Ganping	Cheng		MD	Jinhua People's Hospital	Jinhua, Zhejiang, China	participated in implementing the study	
Han	Yu		MD	YongJia County People's Hospital	Wenzhou, Zhejiang, China	participated in implementing the study	
Lianjiang	Zhong		MD	Tongxiang Second People's Hospital	Jiaxing, Zhejiang, China	participated in implementing the study	
Yongwei	Li		MD	Qingtian People's Hospital	Lishui, Zhejiang, China	participated in implementing the study	
Xiaoping	Sun		MD	Zhenhai District People's Hospital of Ningbo	Ningbo, Zhejiang, China	participated in implementing the study	
Gonghua	Pan		MD	Health Community Group of Yuhuan Second People's Hospital	Taizhou, Zhejiang, China	participated in implementing the study	
Bifeng	Zhong		MD	Zhoushan Putuo District People's Hospital	Zhoushan, Zhejiang, China	participated in implementing the study	
Weimin	Feng		MD	Hangzhou Hospital of Traditional Chinese Medicine	Hangzhou, Zhejiang, China	participated in implementing the study	
Kan	Ouyang		MD	Red Cross Hospital of Hangzhou	Hangzhou, Zhejiang, China	participated in implementing the study	
Jiawei	Li		MD	The Fifth People's Hospital of Yuhang District	Hangzhou, Zhejiang, China	participated in implementing the study	
Yiqing	Jiang		MD	Taizhou Enze Medical Center (Group) Luqiao Hospital	Taizhou, Zhejiang, China	participated in implementing the study	
likang	Lan		MD	Lishui People's Hospital	Lishui, Zhejiang, China	participated in implementing the study	
